# Biodegradable ICG-Conjugated Germanium Nanoparticles for *In Vivo* Near-Infrared Dual-Modality Imaging and Photothermal Therapy

**DOI:** 10.1021/acsami.4c10800

**Published:** 2024-10-24

**Authors:** Guo Chen, Pengbo He, Cui Ma, Jie Xu, Taiyu Su, Jingfei Wen, Hao-Chung Kuo, Lili Jing, Sung-Liang Chen, Chang-Ching Tu

**Affiliations:** † University of Michigan-Shanghai Jiao Tong University Joint Institute, Shanghai Jiao Tong University, Shanghai 200240, China; ‡ Engineering Research Center of Cell & Therapeutic Antibody, Ministry of Education, and School of Pharmacy, Shanghai Jiao Tong University, Shanghai 200240, China; § School of Chemistry and Chemical Engineering, Shanghai Jiao Tong University, Shanghai 200240, China; ∥ Semiconductor Research Center, Foxconn Research, Shenzhen 518109, China; ⊥ Institute of Medical Robotics, Shanghai Jiao Tong University, Shanghai 200240, China; # Engineering Research Center of Digital Medicine and Clinical Translation, Ministry of Education, Shanghai 200030, China; ∇ State Key Laboratory of Advanced Optical Communication Systems and Networks, Shanghai Jiao Tong University, Shanghai 200240, China; ○ Department of Electrical Engineering, National Central University, Taoyuan 320317, Taiwan

**Keywords:** theranostics, biodegradability, fluorescence imaging, photoacoustic imaging, photothermal therapy

## Abstract

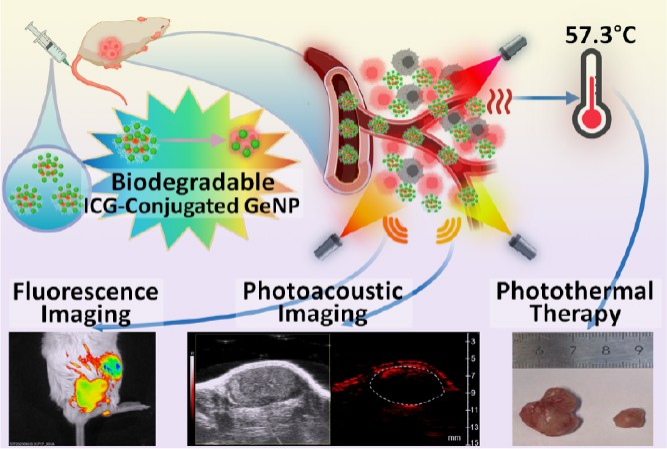

Theranostics, by integrating diagnosis and therapy on a single platform, enables real-time monitoring of tumors during treatment. To improve the accuracy of tumor diagnosis, the fluorescence and photoacoustic imaging modalities can complement each other to achieve high resolution and a deep penetration depth. Despite the superior performance, the biodegradability of theranostic agents plays a critical role in enhancing nanoparticle excretion and reducing chronic toxicity, which is essential for clinical applications. Herein, we synthesize biocompatible and biodegradable indocyanine green (ICG)-conjugated germanium nanoparticles (GeNPs) and investigate their biodistributions in nude mice and 4T1 tumor models after intravenous injections using near-infrared (NIR) dual-modality fluorescence and photoacoustic imaging. The ICG-conjugated GeNPs have strong NIR absorption due to the NIR-absorbing ICG and Ge in combination, emit strong NIR fluorescence due to the multilayered ICG coatings, and exhibit very low *in vitro* and *in vivo* toxicity. After tail vein injections, the ICG-conjugated GeNPs mainly accumulate in the liver and spleen as well as the tumor with the help of the enhanced permeability and retention effect. The tumor’s fluorescence signal is much stronger than that of the control group injected with pure ICG solution, as the GeNPs can function as biodegradable carriers for efficiently delivering the ICG molecules to the tumor. Lastly, the ICG-conjugated GeNPs accumulated in the tumor can also be utilized for photothermal treatment under NIR laser irradiation, after which the tumor volume almost diminishes after 14 days. The experimental findings in this work demonstrate that the ICG-conjugated GeNPs are promising theranostic agents with exceptional biodegradability for *in vivo* NIR dual-modality imaging and photothermal therapy.

## Introduction

Theranostics, which enables the diagnosis and treatment of tumors simultaneously, has shown great potential for personalized oncology.^1−4^ After specific binding to the tumor, the theranostic agents provide not only real-time information about the size and location of the tumor but also a therapeutic effect for treating the tumor.^5,6^ Since there is no time delay and spatial variation between the diagnosis and treatment, the overall therapeutic efficacy can be enhanced. Nanoparticles loaded with medicine can be further conjugated with targeting molecules for achieving simultaneous imaging and precise drug delivery.^7^ For example, iodine-conjugated Pt(IV) nanoparticles can facilitate computed tomography (CT) imaging, while performing chemotherapy of tumors at the same time.^8^ In addition to chemotherapy, gold nanoparticles with mixed-charge zwitterionic surfaces can undergo pH-triggered self-assembly in tumors and enable photothermal therapy, with the process monitored by photoacoustic (PA) imaging.^9^ Similarly, Zn(II)-PPIX-loaded UiO-66 metal–organic framework nanoparticles can function as fluorescent probes for miRNA-guided fluorescence imaging, while providing photodynamic therapy to kill the cancer cells.^10^

Optical imaging, although having the advantages of noninvasiveness and high resolution, is mainly limited by the imaging depth, particularly in the visible range due to tissue scattering and fluorophore reabsorption.^11^ Red-shifting the imaging wavelength from the visible to the near-infrared (NIR) is a widely used method to increase the imaging depth. For example, the *in vivo* fluorescence microscopic imaging of mouse cerebral vasculature in the window of 1400 to 1500 nm has demonstrated an admirable imaging depth of about 1.3 mm.^12^ On the other hand, PA imaging, with the characteristic of laser input and acoustic output, is a hybrid imaging method combining optical and ultrasonic modalities.^13^ By using an NIR excitation wavelength of 1064 nm, the *in vivo* PA microscopic imaging of melanin molecules in tissues with a penetration depth of up to 8 mm has been achieved.^14^ Therefore, it is desirable to combine the complementary advantages of NIR fluorescence and PA imaging, such as high resolution, high contrast, and large penetration depth, into a single platform, thereby increasing cancer diagnosis accuracy. Recently, a great variety of NIR dual-modality fluorescence and PA imaging contrast agents have been developed, such as activatable cyanine-based probes,^15^ PEGylated semiconducting polymer nanoparticles,^16^ indocyanine green (ICG)-conjugated mesoporous silica-coated gold nanobipyramids,^17^ peptide-functionalized carbon dots,^18^ amorphous Ag_2-x_Cu_*x*_S quantum dots,^19^ and gold and dendritic mesoporous silica-encapsulated lanthanide-doped nanoparticles.^20^

Despite the tremendous efforts devoted to the development of theranostic agents and their exceptional imaging and therapeutic efficacy demonstrated, biocompatibility and biodegradability remain the most critical issues for *in vivo* clinical applications.^21,22^ When the nanoparticles are administered intravenously, those smaller than 5 nm exhibit rapid renal excretion.^23^ In comparison, the nanoparticles ranging from 5 to 200 nm have longer circulation times in the bloodstream before being captured by the mononuclear phagocytic system (MPS). However, if the nanoparticle size exceeds 200 nm, they tend to be filtered out by the MPS in a relatively short time, leading to rapid accumulations in the liver and spleen.^24,25^ It is worth noting that the nanoparticles between 30 and 200 nm can be trapped in the tumor lesions with the help of the enhanced permeability and retention (EPR) effect.^26,27^ To be fully removed from the organism after carrying out their functionalities, the biodegradation of the nanoparticles into excretable molecules or ions can help reduce chronic toxicity.^28,29^ Recently, various nanoparticles that can degrade spontaneously in biomedical applications have been reported, such as NIR-absorbing germanium nanoparticles (GeNPs) with *in vivo* degradation time of about 48 h for PA imaging,^30^ polymeric nanocarriers of flavonoids for preventing and treating inflammatory bowel disease,^31^ microenvironment-activated silica nanoplatforms for NIR fluorescence imaging and chemodynamic therapy,^32^ porous silicon nanoparticles for time-gated fluorescence imaging, photodynamic and photothermal therapies,^33^ and metabolically digestible biliverdin nanoparticles for sentinel lymph node detection by PA imaging.^34^

**Scheme 1:**
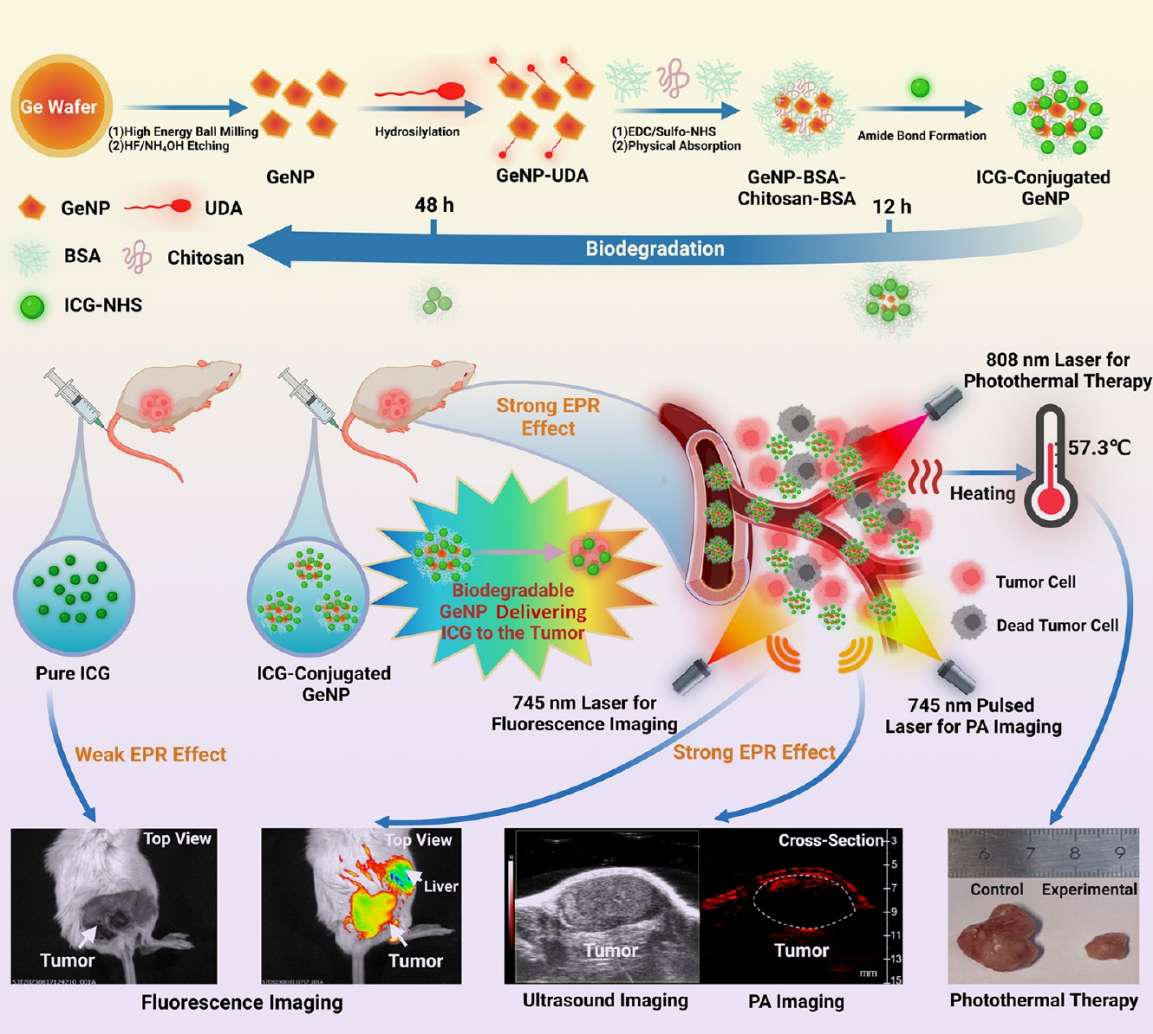
(A) Synthesis and Biodegradation Process of the ICG-Conjugated GeNPs as a Theranostic Platform and (B) *In Vivo* Dual-Modality Fluorescence and PA Imaging and Photothermal Treatment after the ICG-Conjugated GeNPs Accumulate in the Tumor by the EPR Effect Image created using BioRender with permission.

ICG is a Food and Drug Administration (FDA)-approved fluorophore widely used in fluorescence imaging. With its excitation peak at 780 nm and emission peak at 820 nm, which is outside most tissue autofluorescence range, ICG enables deep penetration imaging for several clinical applications, such as retinal angiography and cardiac perfusion.^35,36^ On the other hand, Ge is an indirect bandgap semiconductor with an energy bandgap equal to 0.7 eV, making it well-suited for NIR photothermal applications.^37^ Furthermore, the biodegradability of Ge has been observed in not only nanoparticles but also bioresorbable electronic sensors,^38^ showing the great potential of Ge-based biological applications. In this work, we synthesize biocompatible and biodegradable ICG-conjugated GeNPs and investigate their biodistributions after intravenous injections by using NIR dual-modality fluorescence and PA imaging. The ICG-conjugated GeNPs possess strong NIR absorption due to the NIR-absorbing ICG and Ge in combination and emit strong NIR fluorescence due to the multilayered ICG coatings (Scheme 1A). The *in vitro* and *in vivo* biodegradations of ICG-conjugated GeNPs are confirmed by time-dependent absorbance spectra and time-dependent fluorescence and PA images, respectively. The *in vivo* and *ex vivo* biodistributions in nude mice reveal that the ICG-conjugated GeNPs mainly accumulate in the liver and spleen and the fluorescence and PA signals almost fully decay in 48 h after the intravenous injections. The *in vivo* biodistributions in 4T1 tumor models show that the ICG-conjugated GeNPs can accumulate in the tumors, and the tumor’s fluorescence signal is much stronger than that of the control group injected with pure ICG solution, as the GeNPs can function as biodegradable carriers for efficiently delivering the ICG molecules to the tumor through the EPR effect (Scheme 1B). Lastly, the ICG-conjugated GeNPs accumulated in the tumor can be utilized for not only NIR imaging but also photothermal treatment, during which the average tumor temperature rises up to 57.3 °C and after which the tumor volume almost diminishes over 14 days. The experimental findings in this work demonstrate that the ICG-conjugated GeNPs can function as a powerful theranostic platform for *in vivo* NIR dual-modality fluorescence and PA imaging, while being capable of photothermal therapy. Most importantly, the ICG-conjugated GeNPs are biodegradable after carrying out their functionalities, which significantly reduces the potential long-term toxicity.

## Results and Discussion

### Synthesis and Characterization of the ICG-Conjugated GeNPs

The GeNPs were synthesized by a similar high-energy ball milling method developed previously.^30^ The detailed synthesis process can be found in the Supporting Information. First, crystalline Ge wafer fragments were pulverized into GeNPs by prolonged high-energy ball milling with 3 mm zirconia beads in isopropanol (IPA), followed by etching in NH_4_OH. After washing and dispersion in IPA, the GeNPs with relatively large particle sizes were precipitated by centrifugation, while the remaining GeNPs in the supernatant were collected and then treated with diluted HF to remove surface oxide. Immediately after the HF treatment, the GeNPs were transferred to deoxygenated neat undecylenic acid (UDA) for a heat-induced hydrosilylation reaction to functionalize the GeNP surface with carboxylic acids. Then, the BSA molecules were conjugated to the carboxylated GeNP surface through amide bond formation. To increase the primary amine binding sites for conjugating with more ICG molecules, the BSA-coated GeNPs were sequentially dispersed by sonication in chitosan solution (3 mg mL^–1^ in 1% v/v acetic acid with the pH adjusted to 5) and then BSA solution (10 mg mL^–1^ in water), so that chitosan and BSA molecules were physically adsorbed onto the GeNP surface. Finally, the BSA/chitosan/BSA-coated GeNPs were treated with NHS-functionalized ICG. The resulting ICG-conjugated GeNPs were washed thoroughly to remove freestanding ICG molecules and then uniformly dispersed in 1× PBS for the following experiments.

**Figure 1:**
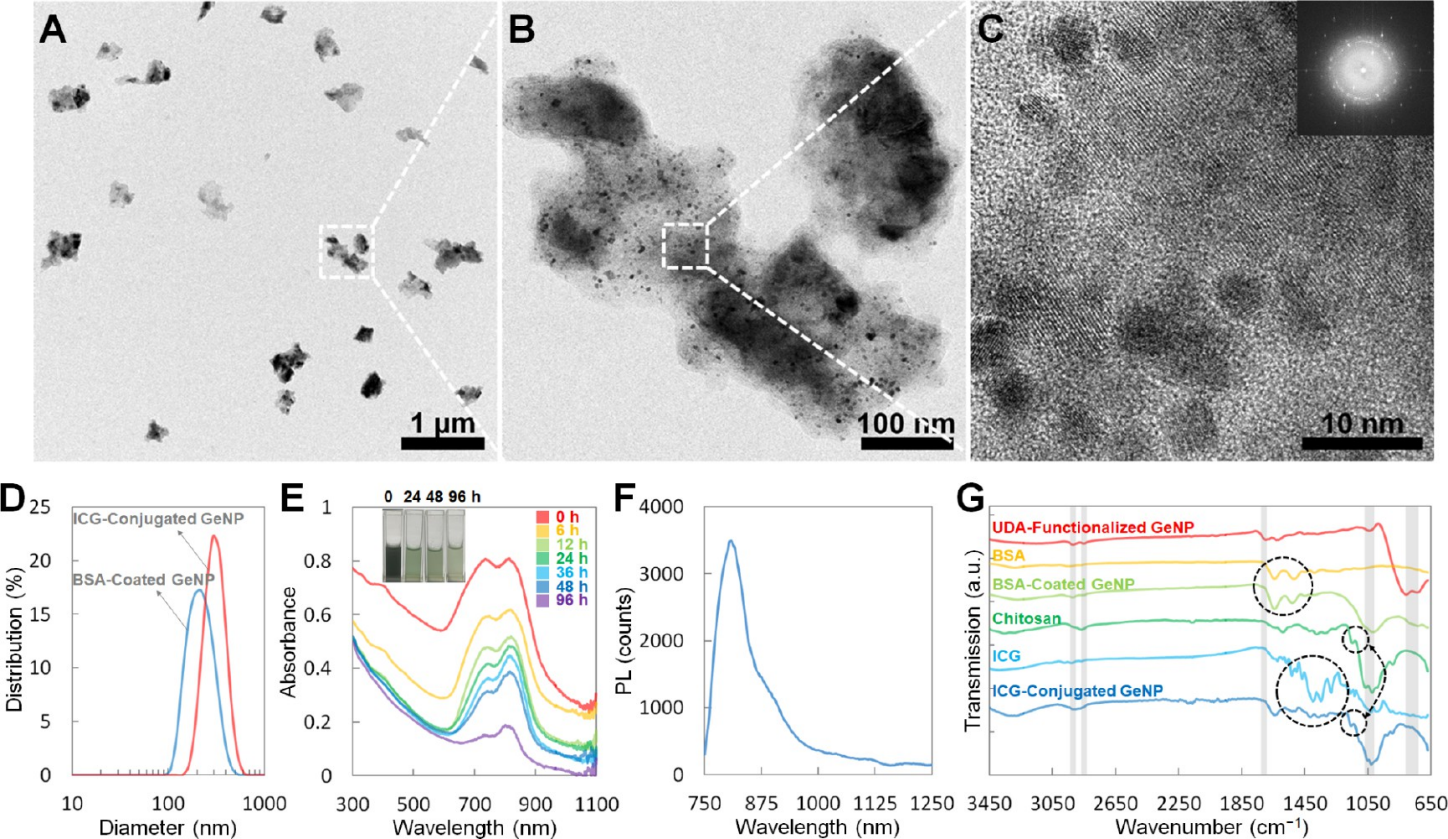
(A–C) TEM images of the ICG-conjugated GeNPs. (D) DLS particle size analysis of the BSA-coated GeNPs (blue line) and the ICG-conjugated GeNPs (red line) in 1× PBS. (E) Absorbance spectra of the ICG-conjugated GeNPs (0.6 mg mL^–1^ in 1× PBS) after degradation times of 0, 6, 12, 24, 36, 48, and 96 h at 37 °C. The inset shows photographs of the ICG-conjugated GeNP suspension color gradually changing from dark gray to light greenish gray as the degradation process continued. (F) PL spectrum of the ICG-conjugated GeNPs in 1× PBS. The PL spectrum was measured with 710 nm excitation. (G) FTIR transmission spectra of the UDA-passivated GeNPs (red line), pure BSA (orange line), BSA-coated GeNPs (light green line), pure chitosan (green line), pure ICG (blue line), and ICG-conjugated GeNPs (purple line). The gray-highlighted absorption peaks, from right to left, correspond to Ge–O–Ge stretching (770 cm^–1^), C–O stretching (1040 cm^–1^), C=O stretching (1710 cm^–1^), and CH_2_ stretching (2850 and 2920 cm^–1^).

As shown in the transmission electron microscopy (TEM) images, the ICG-conjugated GeNPs have sizes around 300 nm (Figure 1A). Furthermore, the crystalline GeNPs, appearing in a dark gray color under TEM, are encapsulated in the BSA/chitosan/BSA-coating, which exhibits a light gray color (Figure 1B). As revealed in the high-resolution TEM image, the GeNP has a nanoporous surface with a porosity less than 5 nm (Figure 1C), which likely results from the NH_4_OH etching step. For comparison, the BSA-coated GeNPs, before the adsorption of chitosan and BSA molecules on the surface, were examined under the TEM, showing smaller particle sizes around 200 nm and similar nanoporous surfaces (Figure S1). The dynamic light scattering (DLS) particle size analysis further confirms that the average hydrodynamic diameter of the BSA-coated GeNPs in 1× PBS is 225.3 nm (blue line in Figure 1D). After the physical adsorption of chitosan and BSA sequentially and the conjugation with ICG, the average hydrodynamic diameter of the ICG-conjugated GeNPs in 1× PBS increases to 313.2 nm (red line in Figure 1D).

The degradation of the ICG-conjugated GeNPs in 1× PBS at 37 °C was monitored by the time-dependent absorbance spectra (Figure 1E). At the beginning of the degradation process (i.e., 0 h), the absorbance spectrum of the ICG-conjugated GeNP suspension (0.6 mg mL^–1^ in 1× PBS) shows two prominent absorption peaks at 745 and 810 nm due to the ICG, which are superimposed on the monotonically decreasing spectrum spanning from UV to NIR due to the GeNPs (Figure S2). For the following experiments, the excitation wavelength of 745 nm is chosen at which the ICG-conjugated GeNPs have the strongest absorption. As the degradation process continued, the absorbance spectrum gradually shifted downward. Accordingly, the appearance of the ICG-conjugated GeNP suspension under white light was dark gray at the beginning and gradually became light greenish gray after 96 h (inset of Figure 1E). Particularly, at 12 h after the degradation process started, the absorbance at 600 nm due to the GeNPs dropped by 69%, while the absorbance at 740 nm due to the conjugated ICG dropped by only 40% (Figure S3A). In other words, the degradation of the crystalline Ge was faster than the conjugated ICG in the first 12 h and then slowed down. Moreover, the increase in the amine binding sites due to the multilayered BSA/chitosan/BSA-coating can also be verified by the time-dependent absorbance spectra (Figure S3B). With only one layer of BSA-coating, although the absorbance spectrum due to the GeNPs is almost identical to the case with the BSA/chitosan/BSA-coating, the two absorbance peaks due to the conjugated ICG are obviously weaker. Furthermore, the ratio of the ICG absorbance at 740 nm to the GeNP absorbance at 600 nm for the case with only one layer of BSA-coating is smaller than that for the case with the BSA/chitosan/BSA-coating.

The degradation of the ICG-conjugated GeNPs was further inspected by scanning electron microscopy energy-dispersive X-ray (SEM-EDX) elemental analysis (Figure S4). As the degradation time increases from 0 to 6 and 24 h, the Ge atomic percentage decreases from 9.9% to 4.4% and 0.9% and the Ge weight percentage also decreases from 37.8% to 21.1% and 4.8%. Moreover, as the crystalline Ge diminishes, the organic layer covering the GeNPs becomes more obvious under SEM. It is worth noting that the ICG-conjugated GeNPs in this work almost fully degrade within 12 h in 1× PBS at 37 °C (Figure 1E), which is much faster than the GeNPs synthesized by a similar high-energy ball milling method but without the NH_4_OH etching step, for which the nearly full degradation time was about 24 h.^30^ The shorter degradation time of the ICG-conjugated GeNPs should be attributed to the nanoporous surface (Figure 1C) caused by NH_4_OH etching. The nanoporous surface leads to a larger surface area and therefore a faster degradation rate. Lastly, the ICG-conjugated GeNPs in 1× PBS exhibit strong NIR photoluminescence (PL) with a peak wavelength of 810 nm (Figure 1F), which is utilized for the following *in vivo* fluorescence imaging.

The surface chemistry of the GeNPs at different stages of the surface modification process was characterized by Fourier transform infrared (FTIR) spectroscopy (Figure 1G). The UDA-functionalized GeNPs (red line in Figure 1G) exhibit absorption peaks of the Ge–O–Ge stretching (770 cm^–1^), C–O stretching (1040 cm^–1^), C=O stretching (1710 cm^–1^), and CH_2_ stretching (2850 and 2920 cm^–1^) attributed to the alkyl chains and carboxylic acids of the UDA ligands. After coating with BSA, the characteristic absorption peaks due to pure BSA between 1450 and 1700 cm^–1^ clearly appear on the absorption spectrum of the BSA-coated GeNPs (light green line in Figure 1G), indicating that the BSA molecules are successfully grafted onto the UDA-functionalized GeNP surface. Lastly, the absorption spectrum of the ICG-conjugated GeNPs (purple line in Figure 1G) features not only the absorption peak of aliphatic ether C–O stretching (1150 cm^–1^) due to chitosan but also the many fine absorption peaks between 1250 and 1550 cm^–1^ due to ICG, confirming that both molecules are successfully grafted on the BSA-coated GeNP surface. In addition, the surface chemistry of the GeNPs was inspected by zeta potential analysis (Figure S5). The zeta potential of the BSA-coated GeNPs was initially −18.4 mV and became −23.5 mV after the physical adsorption of chitosan and BSA molecules. Given that the BSA zeta potential in the literature is about −30 mV at pH 7,^39^ the decrease in zeta potential confirms that more BSA molecules are loaded onto the GeNP surface. Lastly, the zeta potential of the ICG-conjugated GeNPs further decreased to −29.6 mV due to the negatively charged sulfonate groups of the ICG molecules.

### Toxicity Studies of the ICG-Conjugated GeNPs

**Figure 2:**
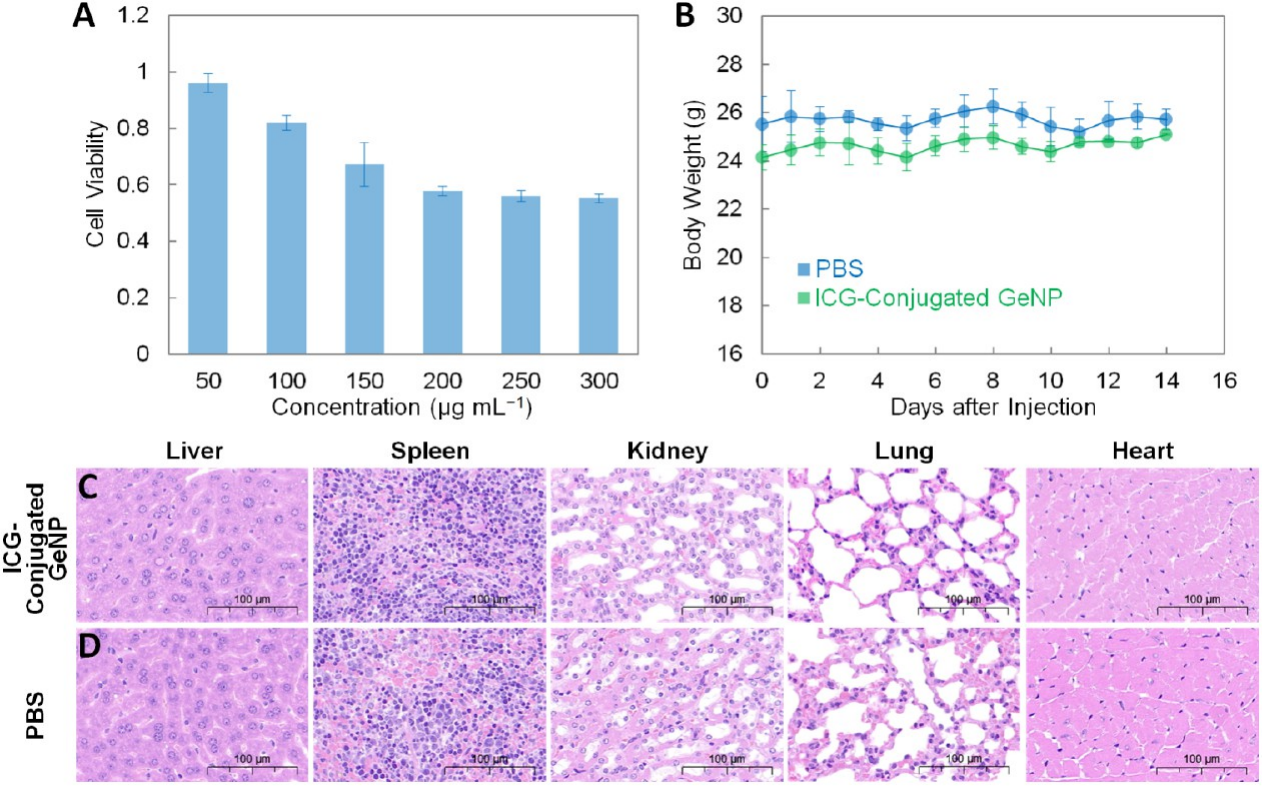
(A) Cell viability assay for the 4T1 cell line treated with ICG-conjugated GeNPs. For a complete assay, three wells per concentration were adopted and the experiment was repeated three times. Each data point represents the mean value of *n* = 3, and the error bar is the standard deviation from the mean. (B) Time-dependent body weight curves for the nude mice injected with the ICG-conjugated GeNP suspension (green line, 150 μL and 3 mg mL^–1^ in 1× PBS) and 1× PBS (blue line, 150 μL), respectively. Each data point represents the mean value of *n* = 3 (i.e., three mice for each data point), and the error bar is the standard deviation from the mean. (C, D) Representative H&E stained images of the major organs of the nude mice injected with the ICG-conjugated GeNP suspension (150 μL, 3 mg mL^–1^ in 1× PBS) and 1× PBS (150 μL).

The *in vitro* cell viability assay for the 4T1 cell line treated with the ICG-conjugated GeNP suspensions with different concentrations (50, 100, 150, 200, 250, and 300 μg mL^–1^ in 1× PBS) was conducted (Figure 2A). Since the fresh ICG-conjugated GeNPs degrade relatively fast in the first 12 h (Figure 1E), which happens during the 48 h cell viability assay period, here, the ICG-conjugated GeNP suspensions after full degradation for 96 h at 37 °C were used for treating the cells, preventing the partial-degraded GeNP residues from adhering to the cells, and causing inaccurate readings of absorption. Based on the assay results, the inhibitory particle concentration, corresponding to 50% cell viability (IC_50_), of the ICG-conjugated GeNPs is estimated to be ≥300 μg mL^–1^, which is comparable to other group-IV nanomaterials, such as the PEG-conjugated Si nanoparticles (IC_50_ ≥ 800 μg mL^–1^), the GeH-PVP nanosheets (IC_50_ ≥ 300 μg mL^–1^), and the natural amine-modified carbon quantum dots (IC_50_ ≥ 500 μg mL^–1^).^40−42^ In addition to the 4T1 tumor cells, normal liver and kidney cells were treated with the ICG-conjugated GeNP suspension, also showing low cytotoxicity up to 300 μg mL^–1^ (Figure S6). Furthermore, the body weight analysis was carried out to evaluate the *in vivo* toxicity (Figure 2B). The ICG-conjugated GeNP suspension (150 μL, 3 mg mL^–1^ in 1× PBS) and 1× PBS (150 μL, as the control group) were intravenously injected into the tail veins of male nude mice (three mice for the ICG-conjugated GeNPs and three mice for PBS), and their body weights were recorded daily for 14 days. Over the 14-day observation period, all mice were raised under the same condition, and no death occurred. Compared to the control group, the mice injected with the ICG-conjugated GeNPs exhibited very similar growing trends in weight. At the end of the observation period, the mice were sacrificed, and their major organs (liver, spleen, kidney, lung, and heart) were collected and fixed in 4% paraformaldehyde. Subsequently, the hematoxylin and eosin (H&E) stained slices were prepared and examined under a microscope (Figure 2C,D). Compared to the control group, the experimental group showed no obvious tissue abnormalities. Lastly, considering that the ICG-conjugated GeNPs were administered through intravenous injections, we took a further step to evaluate hemolysis using zebrafish models. The zebrafish embryos treated with the ICG-conjugated GeNPs by either injection or soaking showed no sign of liver toxicity or hemolysis during the experiment (Figures S7 and S8).

### Concentration-Dependent Fluorescence and PA Imaging of the ICG-Conjugated GeNPs

**Figure 3:**
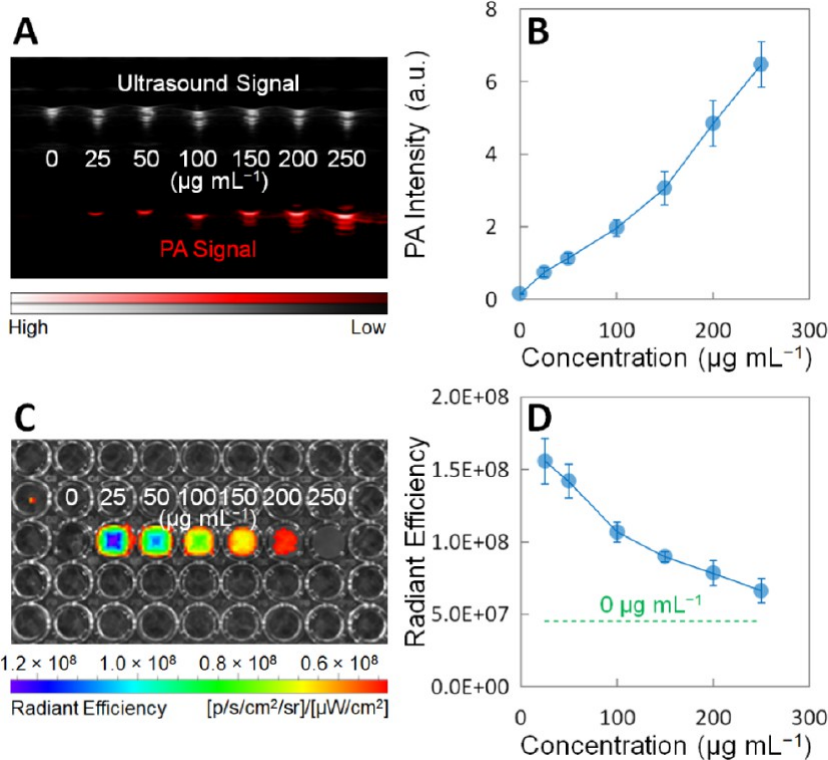
(A, B) Concentration-dependent ultrasound/PA images and corresponding PA intensities of the ICG-conjugated GeNP suspensions with different concentrations (0, 25, 50, 100, 150, 200, and 250 μg mL^–1^ in 1× PBS). The suspensions were loaded into the plastic pipes. (C, D) Concentration-dependent fluorescence images and corresponding radiant efficiencies of the ICG-conjugated GeNP suspensions with different concentrations (0, 25, 50, 100, 150, 200, and 250 μg mL^–1^ in 1× PBS). The suspensions were loaded into the wells of a 96-well culture plate. Each data point in (B) and (D) represents the mean value of *n* = 3 (i.e., three pipes or three wells for each data point), and the error bar is the standard deviation from the mean.

The concentration-dependent efficacies of the ICG-conjugated GeNPs as PA contrast agents and fluorescent probes were investigated (Figure 3). First, the ICG-conjugated GeNP suspensions with different concentrations (0, 25, 50, 100, 150, 200, and 250 μg mL^–1^ in 1× PBS) were loaded into plastic pipes and then placed under the PACT system (VEVO LAZR-X) for PA imaging. As shown in the ultrasound/PA images under excitation at 745 nm, the PA intensity almost linearly increases as the ICG-conjugated GeNP concentration increases from 0 to 250 μg mL^–1^ (Figure 3A,B). In contrast, the ultrasound signal does not change much among different concentrations (upper row in Figure 3A). Second, the ICG-conjugated GeNP suspensions with different concentrations (0, 25, 50, 100, 150, 200, and 250 μg mL^–1^ in 1× PBS) were loaded into different wells of a 96-well culture plate and placed under the IVIS imaging system (PerkinElmer Spectrum) for fluorescence imaging. As shown in the fluorescence images under excitation at 745 nm and emission at 840 nm, the fluorescence radiant efficiency almost linearly decreases as the ICG-conjugated GeNP concentration increases from 25 to 250 μg mL^–1^, and all concentrations exhibit stronger fluorescence than the background (0 μg mL^–1^) (Figure 3C,D). The inverse relationship between the ICG-conjugated GeNP concentration and the fluorescence intensity can be attributed to the fluorescence reabsorption of the GeNPs and ICG molecules.^43^ Finally, the fluorescence stability of ICG-conjugated GeNPs was characterized. After 20 times of consecutive exposures under the IVIS imaging system, the fluorescence intensity of the ICG-conjugated GeNPs only shows slight attenuation due to the photobleaching of ICG (Figure S9).

### Biodistribution by *In Vivo* Fluorescence and PA Imaging

**Figure 4:**
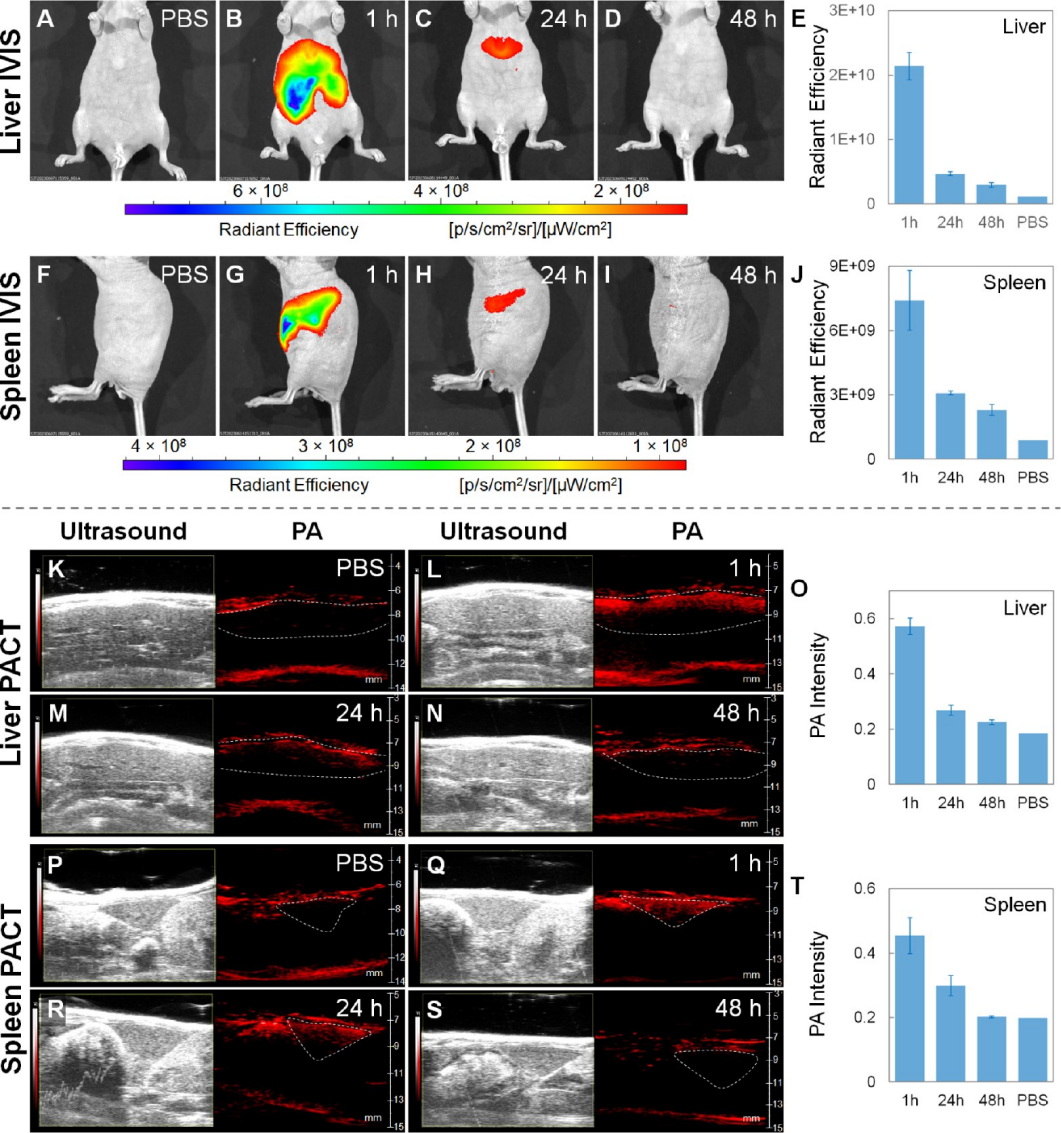
(A–D and F–I) Time-dependent IVIS fluorescence images and (E, J) corresponding radiant efficiencies of the livers and spleens of the nude mice intravenously injected with the ICG-conjugated GeNP suspension (150 μL, 3 mg mL^–1^ in 1× PBS) and 1× PBS (150 μL, as the control group), respectively. (K–N and P–S) Time-dependent ultrasound/PA images and (O, T) corresponding PA intensities of the livers and spleens of the nude mice intravenously injected with the ICG-conjugated GeNP suspension (150 μL, 3 mg mL^–1^ in 1× PBS) and 1× PBS (150 μL, as the control group), respectively. Note that (B–D), (G–I), (L–N), and (Q–S) belong to one mouse (experimental group) and (A), (F), (K), and (P) belong to another mouse (control group). The images were taken at 1, 24, and 48 h after the intravenous injection. Each data point in (E), (J), (O), and (T) represents the mean value of *n* = 3 (i.e., three mice for each data point), and the error bar is the standard deviation from the mean.

Next, we investigated the biodistributions in nude mice, which were intravenously injected with the ICG-conjugated GeNP suspension (150 μL, 3 mg mL^–1^ in 1× PBS) and 1× PBS (150 μL, as the control group), respectively, using *in vivo* fluorescence and ultrasound/PA imaging at different time points (1, 24, and 48 h) after the injection (Figure 4). For each time point, fluorescence imaging was first conducted using the IVIS imaging system with excitation at 745 nm and emission at 840 nm. Then, the mouse was moved to the PACT system for ultrasound/PA imaging with excitation at 745 nm. In addition to imaging, the time-dependent radiant efficiencies and PA intensities at the organ regions of interest are also shown. At 1 h after the injection, both the liver and spleen exhibited strong fluorescence and PA signals. At 48 h after the injection, the fluorescence and PA signals in the liver were reduced by 86% and 61%, respectively, while the fluorescence and PA signals in the spleen were reduced by 69% and 55%, respectively. The decay trends of the fluorescence and PA signals are consistent with each other. With the help of the ultrasound mode, which clearly delineates the morphology of each organ, we can see that the PA signals are mostly located close to the upper surface, which should be attributed to tissue scattering of the excitation light, limiting penetration depth in PA imaging. Compared to the control group injected with pure ICG, only the mouse injected with the ICG-conjugated GeNP suspension shows fluorescence signals at the liver and spleen (Figures S10 and S11). These results can be explained by the fact that the ICG-conjugated GeNPs have relatively large particle sizes and therefore tend to accumulate in MPS organs, such as the liver and spleen, more efficiently than the ICG molecules. It is worth mentioning that the pure ICG with a concentration of 0.025 μg mL^–1^ exhibits stronger fluorescence than the ICG-conjugated GeNPs with a concentration of 25 μg mL^–1^ under the IVIS imaging system (Figure S12). Therefore, the volume and concentration of the pure ICG solution (150 μL, 50 μg mL^–1^ in 1× PBS) injected into the mouse should be sufficient to make a fair comparison with the ICG-conjugated GeNP suspension (150 μL, 3 mg mL^–1^ in 1× PBS) for the time-dependent *in vivo* fluorescence imaging investigations as shown in Figures S10, S11, and S14.

### Biodistribution by *Ex Vivo* Fluorescence and PA Imaging

**Figure 5:**
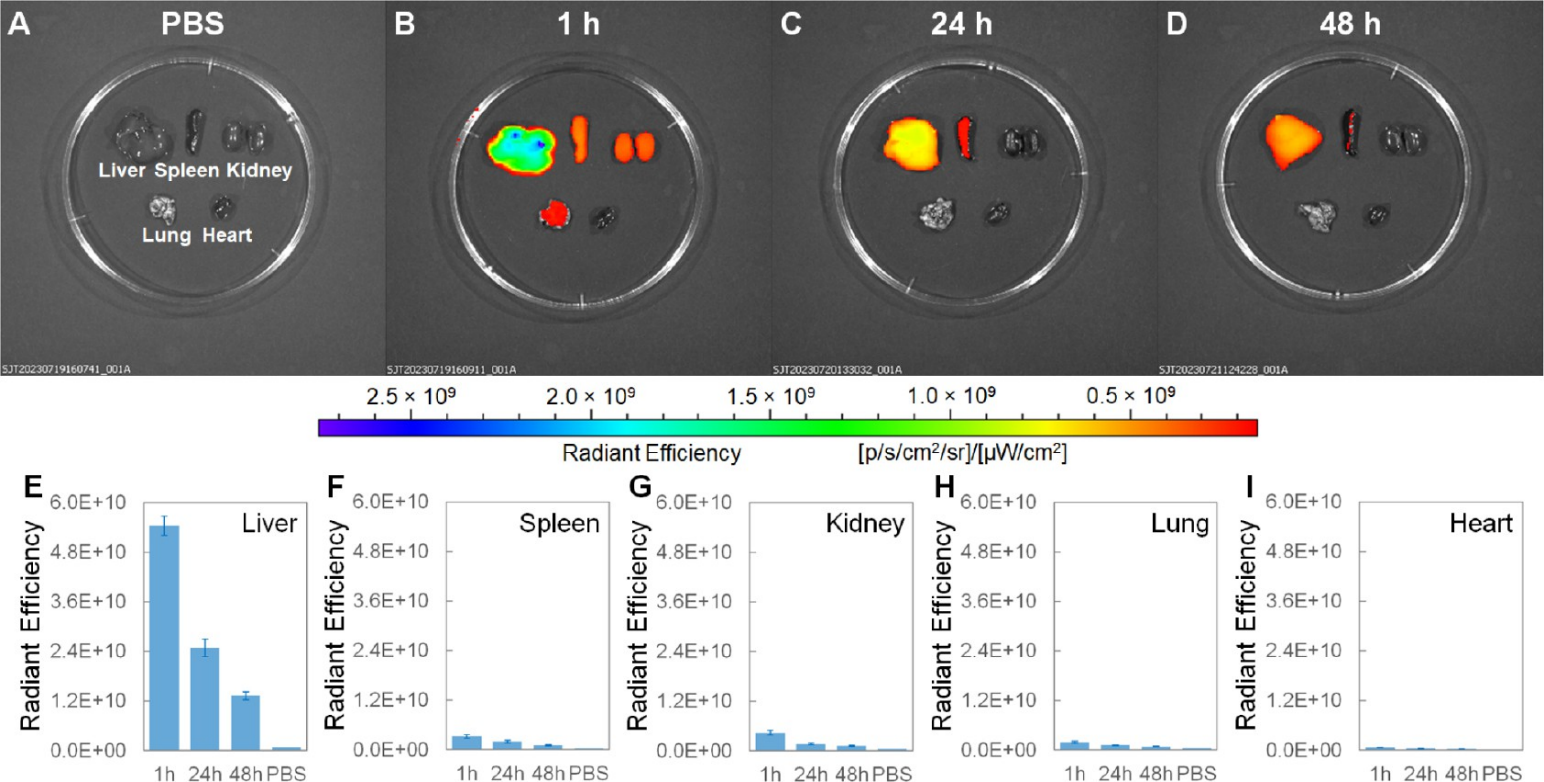
(A–D) Time-dependent IVIS fluorescence images and (E–I) corresponding radiant efficiencies of the major organs (liver, spleen, kidney, lung, and heart) collected from the male BALB/c mice intravenously injected with the ICG-conjugated GeNP suspension (150 μL, 3 mg mL^–1^ in 1× PBS) and 1× PBS (150 μL, as the control group), respectively. Note that the major organs in (A) belong to one mouse (control group) and the major organs in (B–D) belong to three mice sacrificed at 1, 24, and 48 h after the intravenous injection, respectively (experimental group). Each data point in (E–I) reflects the mean value of *n* = 3 (i.e., three mice for each data point), and the error bar is the standard deviation from the mean.

**Figure 6:**
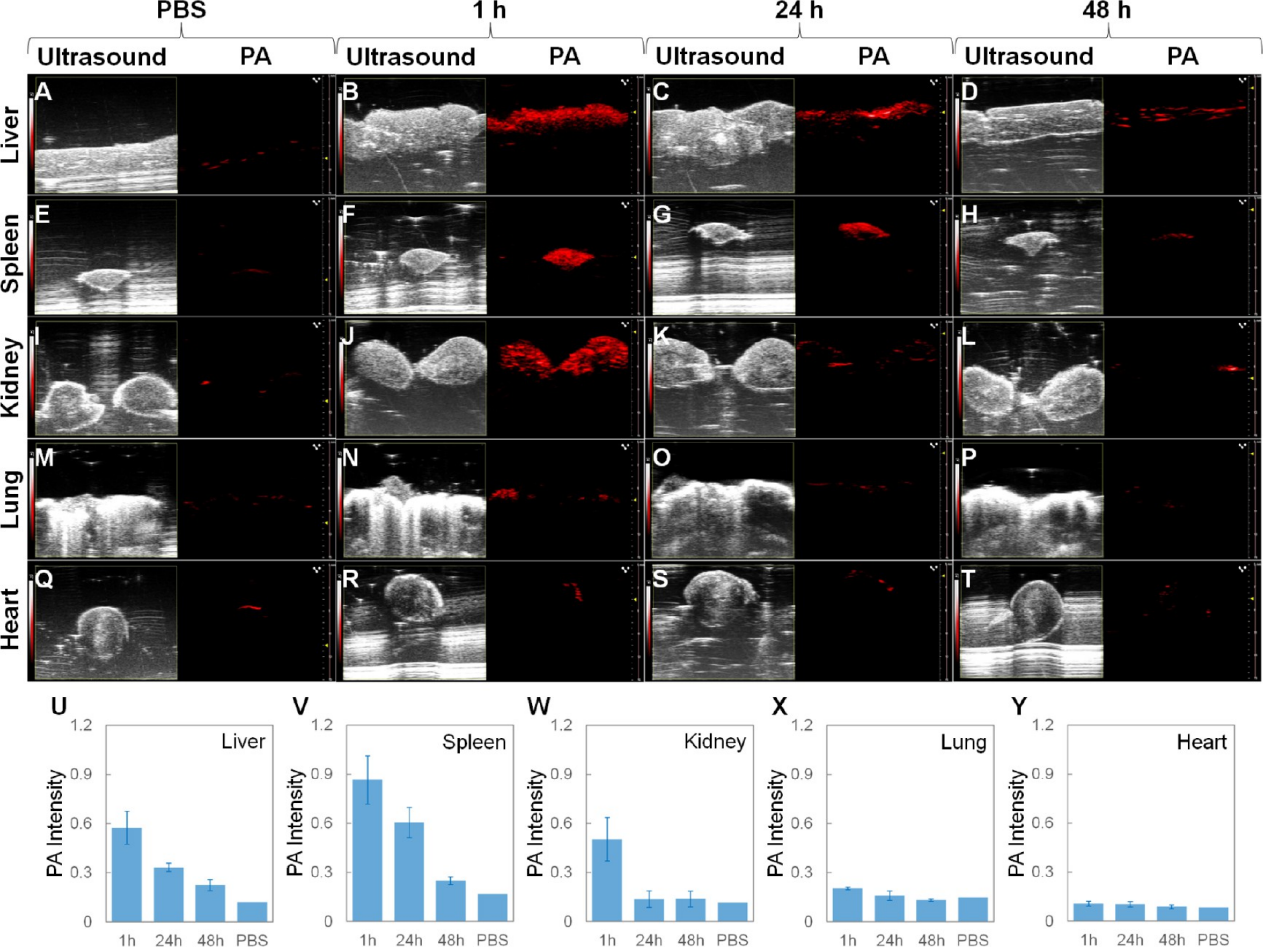
(A–T) Time-dependent ultrasound/PA images and (U–Y) corresponding PA intensities of the major organs (liver, spleen, kidney, lung, and heart) collected from the male BALB/c mice intravenously injected with the ICG-conjugated GeNP suspension (150 μL, 3 mg mL^–1^ in 1× PBS) and 1× PBS (150 μL, as the control group), respectively. Note that the major organs in the PBS column belong to one mouse (control group) and the major organs in the 1, 24, and 48 h columns belong to three mice sacrificed at different time points after the intravenous injection (experimental group). Each data point in (U–Y) reflects the mean value of *n* = 3 (i.e., three mice for each data point), and the error bar is the standard deviation from the mean.

To further confirm the biodistributions shown in Figure 4, *ex vivo* fluorescence imaging (Figure 5) and *ex vivo* ultrasound/PA imaging (Figure 6) were carried out. The male BALB/c mice were intravenously injected with the ICG-conjugated GeNP suspension (150 μL, 3.0 mg mL^–1^ in 1× PBS) and 1× PBS (150 μL, as the control group), respectively. At different time points (1, 24, and 48 h) after the injection, the mice were sacrificed and their major organs (liver, spleen, kidney, lung, and heart) were dissected and immediately subjected to IVIS fluorescence imaging with excitation at 745 nm and emission at 840 nm. The time-dependent fluorescence images show that at 1 h after the injection, the fluorescence signal at the liver was much stronger than at the spleen, kidney, lung, and heart. At 48 h after the injection, the liver fluorescence signal was reduced by 76%, while the fluorescence signals at all other organs almost decayed to the PBS level. Right after each fluorescence imaging, the major organs were moved to the PACT system for ultrasound/PA imaging with an excitation wavelength of 745 nm. At 1 h after the injection, the spleen PA signal was stronger than that of the liver. At 48 h after the injection, the spleen and liver PA signals were reduced by 71% and 61%, respectively. Meanwhile, the PA signals at all other organs were indistinguishable from those at the PBS level. Overall, the *ex vivo* signal decaying trends here are consistent with the *in vivo* signal decaying trends as shown in Figure 4.

### *In Vivo* Fluorescence and PA Imaging on 4T1 Tumor Models

**Figure 7:**
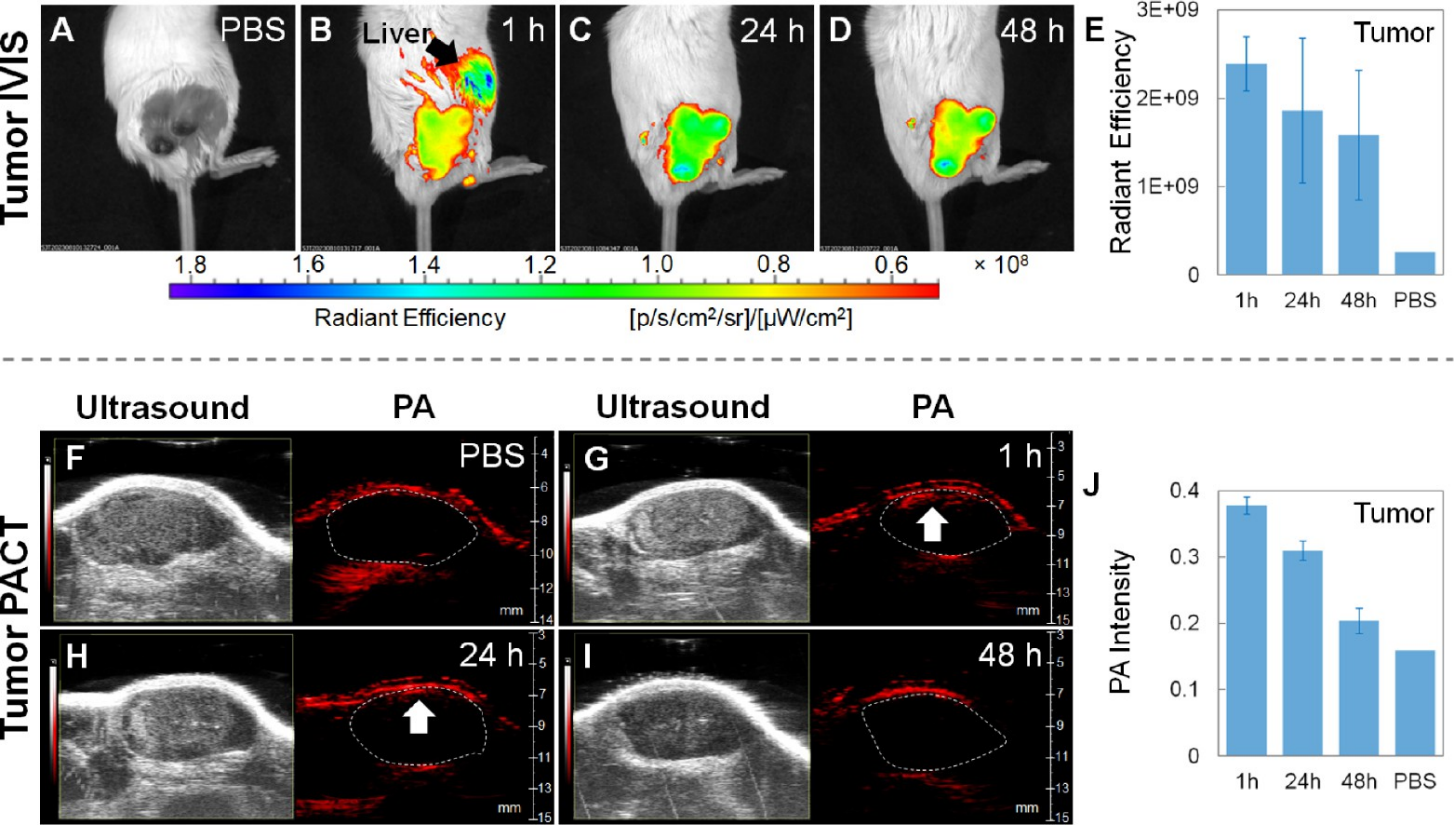
(A–D) Time-dependent IVIS fluorescence images and (E) corresponding radiant efficiencies of the 4T1 tumors of the mouse models intravenously injected with the ICG-conjugated GeNP suspension (150 μL, 3 mg mL^–1^ in 1× PBS) and 1× PBS (150 μL, as the control group), respectively. (F–I) Time-dependent ultrasound/PA images and (J) corresponding PA intensities of the 4T1 tumors of the mouse models intravenously injected with the ICG-conjugated GeNP suspension (150 μL, 3 mg mL^–1^ in 1× PBS) and 1× PBS (150 μL, as the control group), respectively. Note that (B–D) and (G–I) belong to one mouse (experimental group), and (A) and (F) belong to another mouse (control group). The images were taken at 1, 24, and 48 h after the intravenous injection. Each data point in (E) and (J) represents the mean value of *n* = 3 (i.e., three mice for each data point), and the error bar is the standard deviation from the mean.

After the biodistribution investigations, the *in vivo* fluorescence and PA dual-modality imaging on 4T1 tumor models was demonstrated (Figure 7). The tumor models were intravenously injected with the ICG-conjugated GeNP suspension (150 μL, 3.0 mg mL^–1^ in 1× PBS) and 1× PBS (150 μL, as the control group), respectively. At different time points (1, 24, and 48 h) after the injection, IVIS imaging with excitation at 745 nm and emission at 840 nm and PACT imaging with the excitation wavelength of 745 nm were conducted for each mouse individually. The time-dependent fluorescence images reveal that at 1 h after the injection (Figure 7B), both tumor and liver exhibited strong fluorescence signals. The tumor signal should be due to the EPR effect, in which the ICG-conjugated GeNPs pass through the leaky blood vessels and become trapped in the tumor tissues.^26,27^ It is worth noting that after incubating the 4T1 cells with the ICG-conjugated GeNPs for 1 and 4 h, minor cellular uptake was observed by utilizing the NIR fluorescence of ICG under a high content screening (HCS) tool equipped with a Cy7 dye observation channel. The 4 h incubation leads to more obvious cellular uptake than 1 h (Figure S13). In comparison, the liver signal should result from the ICG-conjugated GeNPs being filtered by the MPS organs, which is also observed in the *in vivo* and *ex vivo* biodistributions shown in Figures 4–6. At 24 and 48 h after the injection (Figure 7C,D), the tumor fluorescence signals were only slightly decreased by 22% and 34%, respectively. However, the liver fluorescence signal totally disappeared because of the biodegradation of the GeNPs. On the other hand, the time-dependent ultrasound/PA images show a similar decaying trend as the fluorescence signals. At 1 h after injection (Figure 7G), the PA signal at the upper part of the tumor near the skin was obviously stronger than the control group (Figure 7F). At 24 and 48 h after the injection (Figure 7H,I), the tumor PA signals were decreased by 18% and 46%, respectively. Lastly, compared to injection with pure ICG, the tumor model injected with the ICG-conjugated GeNPs exhibited much stronger fluorescence signals at all times after the injection (Figure S14). It demonstrates that the GeNPs can serve as biodegradable carriers for efficiently delivering the ICG molecules to the tumor by taking advantage of the EPR effect. Although the relatively large particle size also leads to accumulation in the liver, the biodegradability of the GeNPs allows the ICG molecules to be cleared from the liver in 24 h.

### Photothermal Therapy on 4T1 Tumor Models

The photothermal property of the ICG-conjugated GeNPs can be applied not only for PA imaging but also for photothermal therapy. First, the photothermal property of the ICG-conjugated GeNPs was investigated by monitoring the temperature variation under 808 nm continuous-wave laser irradiation (1 W cm^–2^) for 10 min. The temperatures and thermographic maps of the ICG-conjugated GeNPs with different concentrations (25, 50, 100, and 150 μg mL^–1^) loaded into different wells of a 96-well culture plate were recorded using an infrared camera every 1 min for 10 min. The temperature of the ICG-conjugated GeNP suspension with a concentration of 150 μg mL^–1^ could be raised to 49.4 °C under the laser irradiation for 10 min, while the PBS as a control displayed negligible temperature increment under the same laser irradiation condition (Figure S15A,B). The temperature of the ICG-conjugated GeNP suspension could be further increased to 54.7 °C as the irradiation power increased to 1.5 W cm^–2^ (Figure S15C). By turning the laser on and off alternately, the photothermal stability of the ICG-conjugated GeNPs was tested in four heating/cooling cycles (Figure S15D). Due to the degradation of the ICG-conjugated GeNPs, the amount of temperature increment in a cycle gradually decreased. Following a heat transfer model described in the literature,^44^ the photothermal conversion efficiency of the ICG-conjugated GeNP suspension (150 μg mL^–1^) was measured to be 51%, which is comparable to other GeNPs synthesized by a laser ablation method (Figure S16).^45^ Next, the *in vitro* photothermal treatment efficacy on the 4T1 cell line was investigated using the cell viability assay (Figure S17). The ICG-conjugated GeNPs showed high biocompatibility without laser irradiation. In contrast, with laser irradiation, the cell viability significantly decreased as the concentration increased. Particularly, the cell viability of the 4T1 cell line under 808 nm laser irradiation for 10 min was only 32% at the ICG-conjugated GeNP concentration of 300 μg mL^–1^. Moreover, the photothermal cytotoxicity of the ICG-conjugated GeNPs on the 4T1 cells was verified using a cell apoptosis assay (Figure S18). In general, the proportion of apoptotic cells increased as the ICG-conjugated GeNP concentration increased. Most importantly, the 808 nm laser irradiation for 10 min obviously increased the apoptosis rate.

**Figure 8:**
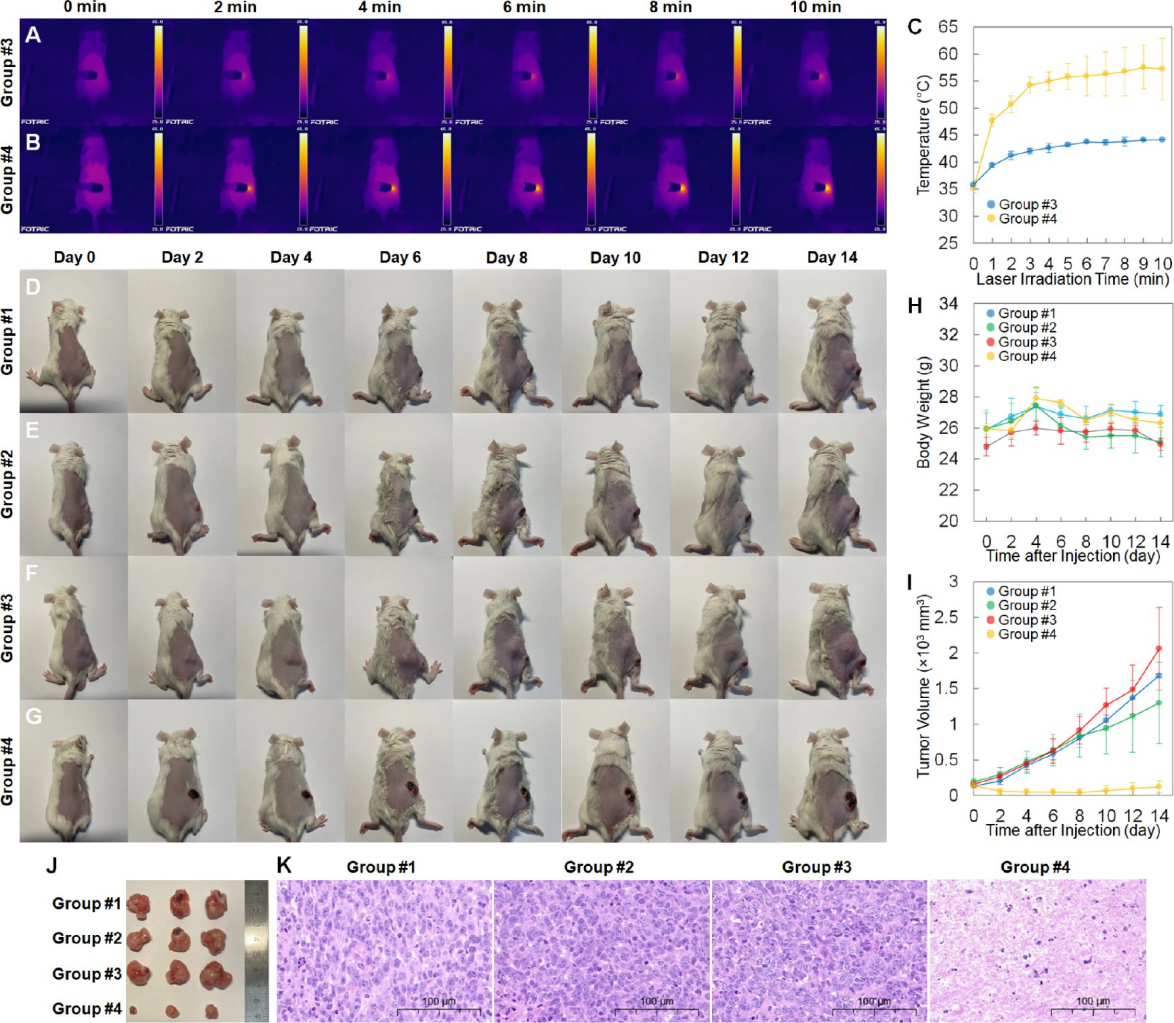
(A and B) Time-dependent infrared thermographic maps and (C) tumor temperatures of the 4T1 tumor models from groups #3 and #4 during the photothermal treatment. (D–G) Time-dependent photographs, (H) body weight curves, and (I) tumor volumes of the 4T1 tumor models from groups #1, #2, #3, and #4 after the photothermal treatment. The data were recorded every 2 days for a total of 14 days. Each data point in (C), (H), and (I) represents the mean value of *n* = 3 (i.e., three mice for each data point), and the error bar is the standard deviation from the mean. (J) Photographs and (K) representative H&E stained images of the tumors dissected from groups #1, #2, #3, and #4 at the end of the 14-day observation period.

Finally, as the volumes of the 4T1 tumor xenografts reached about 150 mm^3^, 12 tumor-bearing mice were randomly divided into 4 groups (*n* = 3 per group): #1 intravenously injected with 1× PBS (150 μL) but without irradiation, #2 intravenously injected with the ICG-conjugated GeNP suspension (150 μL, 3 mg mL^–1^ in 1× PBS) but without irradiation, #3 no injection but with NIR irradiation, and #4 intravenously injected with the ICG-conjugated GeNP suspension (150 μL, 3 mg mL^–1^ in 1× PBS) but with NIR irradiation. The groups #1, #2, and #3 serve as the control group, while the group #4 serves as the experimental group. At 1 h after the injection, the mouse was anesthetized, and then, the NIR irradiation was performed, with the entire region of the tumor irradiated by an 808 nm laser (1 W cm^–2^) for 10 min. To monitor the *in vivo* photothermal effect, the temperatures of the tumor and the thermographic maps of the mice were recorded using an infrared camera (Figure S19). Under the NIR laser irradiation, the average tumor temperature of the mice injected with the ICG-conjugated GeNPs (group #4) can reach 57.3 °C, which is sufficient for tumor ablation. In contrast, the average tumor temperature of the control group injected with PBS (group #3) can attain only 44.1 °C (Figure 8A–C). After the photothermal treatment, the tumor appearances (Figure 8D–G), the body weights (Figure 8H), and the 4T1 tumor volumes (Figure 8I) of the mice in different groups were recorded every 2 days for a total of 14 days. Here, the tumor volume, measured by a caliper, was estimated using the formula: (tumor length) × (tumor width)^2^/2. No mouse showed a significant reduction in body weight during the 14 days of observation, indicating that these treatments have negligible adverse effects on the mice. Furthermore, the tumor volumes of the mice in groups #1, #2, and #3 constantly increased, growing to about 1500 mm^3^ in 14 days. In contrast, the tumors in group #4 gradually decreased in size, being almost obliterated in 8 days after the photothermal treatment. Although there was still slight recurrence later on, the final tumor volumes were suppressed to below 100 mm^3^. It is worth noting that a scar was left on the tumor surface after the photothermal treatment, which was commonly found in other photothermal therapy studies.^46,47^ Lastly, after the 14-day observation, the mice from each group were sacrificed and their tumors were dissected and H&E stained (Figure 8J,K). The tumors from group #4 exhibit significantly smaller sizes than those from other groups, and a large amount of cell necrosis and nuclear lysis appears in the dissected tumor from group #4, both of which provide strong evidence that photothermal treatment using the ICG-conjugated GeNPs can achieve tumor ablation and inhibit tumor growth. To highlight the importance of the EPR effect, which helps the ICG-conjugated GeNPs deliver ICG molecules to the tumor, additional three tumor-bearing mice were injected with pure ICG solution (150 μL, 50 μg mL^–1^ in 1× PBS) and then subjected to the same analysis as mentioned above (Figure S20). The results show that the average tumor temperature could only attain 44.5 °C, which is similar to that of group #3, and the tumor volume constantly increased after the treatment. The poor photothermal effect of pure ICG is due to its rapid uptake by the liver and insufficient accumulation in the tumor.^48^ Lastly, while the EPR effect plays a significant role in the photothermal therapy, as shown by the comparison between group #4 in Figure 8I (with EPR) and Figure S20E (without EPR), coupling the ICG-conjugated GeNPs with targeting biomolecules will be a critical step to further improve the accumulation efficiency in the tumor.

## Conclusion

In summary, we investigated the biodistributions of biodegradable ICG-conjugated GeNPs in nude mice and 4T1 tumor models using NIR dual-modality fluorescence and PA imaging. We also confirm the photothermal therapy efficacy of the ICG-conjugated GeNPs, which accumulate in the tumor through the EPR effect. The ICG-conjugated GeNPs were synthesized by high-energy ball milling and etching, followed by bioconjugation with BSA, chitosan, and BSA sequentially and functionalization with ICG via amide bond formation. The ICG-conjugated GeNPs possess strong NIR absorption, which is attributed to the NIR-absorbing ICG and Ge in combination, and emit strong NIR fluorescence, which is enhanced by the multilayered ICG-functionalized coatings. As revealed by the cell viability assay (IC_50_ ≥ 300 μg mL^–1^), 14-day body weight analysis, and histological images of the major organs, the ICG-conjugated GeNPs have very low *in vitro* and *in vivo* toxicity. The *in vitro* and *in vivo* biodegradation properties of the ICG-conjugated GeNPs are confirmed by the time-dependent absorbance spectra in 1× PBS and the time-dependent fluorescence and PA images of the major MPS organs, respectively. The NIR dual-modality imaging analysis shows that at 1 h after the intravenous injection, both the liver and spleen exhibit strong fluorescence and PA signals, and the signals almost fully decay in 48 h. The ICG-conjugated GeNPs can also accumulate in the 4T1 tumor after blood circulation, and the tumor’s fluorescence signal is much stronger than that of the control group injected with pure ICG solution, demonstrating that the GeNPs can function as biodegradable carriers for efficiently delivering the ICG molecules to the tumor through the EPR effect. Finally, under NIR laser irradiation, the ICG-conjugated GeNPs accumulated in the tumor can elevate the average tumor temperature to 57.3 °C, compared to 44.1 °C attained by the control group injected with PBS. Besides, the tumor volume of the experimental group almost fully diminishes in 14 days, and a significant amount of cell necrosis and nuclear lysis is observed in the dissected tumor. In comparison, the tumor volumes of the control groups kept growing for 14 days. The experimental findings in this work demonstrate that the ICG-conjugated GeNPs are biocompatible and biodegradable theranostic agents for *in vivo* NIR dual-modality fluorescence and PA imaging, as well as photothermal therapy.

## Supporting Information Available

TEM images of the BSA-coated GeNPs; absorbance spectrum of the BSA-coated GeNP suspension; absorbance spectra of the ICG-conjugated GeNPs with three-layered BSA/chitosan/BSA-coating and only one-layered BSA-coating; SEM images and EDX elemental analyses of the ICG-conjugated GeNPs during the *in vitro* degradation process; zeta potentials of the BSA-coated GeNPs, BSA/chitosan/BSA-coated GeNPs, and ICG-conjugated GeNPs; cell viability assay for the normal liver and kidney cells treated with the ICG-conjugated GeNPs; toxicity and hemolysis tests of the ICG-conjugated GeNPs on zebrafish embryos; photostability of the ICG-conjugated GeNPs under the IVIS imaging system; time-dependent IVIS fluorescence images of the livers, spleens, and tumors with the injections of the ICG-conjugated GeNP suspension and pure ICG solution; pure ICG solution dose calibration under the IVIS imaging system; *in vitro* cellular uptake of the ICG-conjugated GeNPs; *in vitro* photothermal properties of the ICG-conjugated GeNPs; estimation of the photothermal conversion efficiency of the ICG-conjugated GeNPs; cell viability assay and apoptosis assay for the 4T1 cell line treated with the ICG-conjugated GeNPs under 808 nm laser irradiation; schematic diagram of the photothermal treatment; photothermal treatment efficacy of pure ICG; experimental details about synthesis of the ICG-conjugated GeNPs, preparation of animal models, cell viability assay, body weight analysis, histological imaging, biodistributions by *in vivo* and *ex vivo* dual-modality imaging, *in vivo* dual-modality imaging of 4T1 tumor models, and characterizations (PDF)

## References

[ref1] Zhang J.; Yin X.; Li C.; Yin X.; Xue Q.; Ding L.; Ju J.; Ma J.; Zhu Y.; Du D.; et al. A Multifunctional Photoacoustic/Fluorescence Dual-Mode-Imaging Gold-Based Theranostic Nanoformulation without External Laser Limitations. Adv. Mater. 2022, 34 (19), 2110690 10.1002/adma.202110690. 35275432

[ref2] Arnold C. Theranostics could be Big Business in Precision Oncology. Nat. Med. 2022, 28, 606–608. 10.1038/s41591-022-01759-6. 35440719

[ref3] Zou Q.; Abbas M.; Zhao L.; Li S.; Shen G.; Yan X. Biological Photothermal Nanodots Based on Self-Assembly of Peptide-Porphyrin Conjugates for Antitumor Therapy. J. Am. Chem. Soc. 2017, 139, 1921–1927. 10.1021/jacs.6b11382. 28103663

[ref4] Chang R.; Zou Q.; Zhao L.; Liu Y.; Xing R.; Yan X. Amino-Acid-Encoded Supramolecular Photothermal Nanomedicine for Enhanced Cancer Therapy. Adv. Mater. 2022, 34 (16), 2200139 10.1002/adma.202200139. 35178775

[ref5] Meng X.; Pang X.; Zhang K.; Gong C.; Yang J.; Dong H.; Zhang X. Recent Advances in Near-Infrared-II Fluorescence Imaging for Deep-Tissue Molecular Analysis and Cancer Diagnosis. Small 2022, 18 (31), 2202035 10.1002/smll.202202035. 35762403

[ref6] Johnson K. K.; Koshy P.; Yang J.; Sorrell C. C. Preclinical Cancer Theranostics—From Nanomaterials to Clinic: The Missing Link. Adv. Funct. Mater. 2021, 31 (43), 2104199 10.1002/adfm.202104199.

[ref7] Manzari M. T.; Shamay Y.; Kiguchi H.; Rosen N.; Scaltriti M.; Heller D. A. Targeted Drug Delivery Strategies for Precision Medicines. Nat. Rev. Mater. 2021, 6, 351–370. 10.1038/s41578-020-00269-6. 34950512 PMC8691416

[ref8] Yu J.; He X.; Zhang Q.; Zhou D.; Wang Z.; Huang Y. Iodine Conjugated Pt(IV) Nanoparticles for Precise Chemotherapy with Iodine-Pt Guided Computed Tomography Imaging and Biotin-Mediated Tumor-Targeting. ACS Nano 2022, 16, 6835–6846. 10.1021/acsnano.2c01764. 35412302

[ref9] Zhang R.; Wang L.; Wang X.; Jia Q.; Chen Z.; Yang Z.; Ji R.; Tian J.; Wang Z. Acid-Induced In Vivo Assembly of Gold Nanoparticles for Enhanced Photoacoustic Imaging-Guided Photothermal Therapy of Tumors. Adv. Healthcare Mater. 2020, 9 (14), 2000394 10.1002/adhm.202000394. 32543023

[ref10] Zhang P.; Ouyang Y.; Sohn Y. S.; Fadeev M.; Karmi O.; Nechushtai R.; Stein I.; Pikarsky E.; Willner I. miRNA-Guided Imaging and Photodynamic Therapy Treatment of Cancer Cells Using Zn(II)-Protoporphyrin IX-Loaded Metal-Organic Framework Nanoparticles. ACS Nano 2022, 16, 1791–1801. 10.1021/acsnano.1c04681. 35020370 PMC8867907

[ref11] Chen Y.; Wang S.; Zhang F. Near-Infrared Luminescence High-Contrast in Vivo Biomedical Imaging. Nat. Rev. Bioeng. 2023, 1, 60–78. 10.1038/s44222-022-00002-8.

[ref12] Feng Z.; Tang T.; Wu T.; Yu X.; Zhang Y.; Wang M.; Zheng J.; Ying Y.; Chen S.; Zhou J.; et al. Perfecting and Extending the Near-Infrared Imaging Window. Light: Sci. Appl. 2021, 10 (1), 197 10.1038/s41377-021-00628-0. 34561416 PMC8463572

[ref13] Zhang X.; Wu Y.; Chen L.; Song J.; Yang H. Optical and Photoacoustic Imaging in Vivo: Opportunities and Challenges. Chem. Biomed. Imaging 2023, 1, 99–109. 10.1021/cbmi.3c00009. 39474621 PMC11504558

[ref14] Wang C.; Guo L.; Wang G.; Ye T.; Wang B.; Xiao J.; Liu X. In-Vivo Imaging of Melanoma with Simultaneous Dual-Wavelength Acoustic-Resolution-Based Photoacoustic/Ultrasound Microscopy. Appl. Opt. 2021, 60, 3772–3778. 10.1364/AO.412609. 33983310

[ref15] Zou X.; Zhao Y.; Lin W. Photoacoustic/Fluorescence Dual-Modality Cyanine-Based Probe for Real-Time Imaging of Endogenous Cysteine and in Situ Diagnosis of Cervical Cancer in Vivo. Anal. Chim. Acta 2023, 1239, 340713 10.1016/j.aca.2022.340713. 36628718

[ref16] Li D.; Zhang C.; Tai X.; Xu D.; Xu J.; Sun P.; Fan Q.; Cheng Z.; Zhang Y. 1064 nm Activatable Semiconducting Polymer-based Nanoplatform for NIR-II Fluorescence/NIR-II Photoacoustic Imaging Guided Photothermal Therapy of Orthotopic Osteosarcoma. Chem. Eng. J 2022, 445, 136836 10.1016/j.cej.2022.136836.

[ref17] Li C.; Mei E.; Chen C.; Li Y.; Nugasur B.; Hou L.; Ding X.; Hu M.; Zhang Y.; Su Z.; Lin J.; Yang Y.; Huang P.; Li Z. Gold-Nanobipyramid-Based Nanotheranostics for Dual-Modality Imaging-Guided Phototherapy. ACS Appl. Mater. Interfaces 2020, 12, 12541–12548. 10.1021/acsami.0c00112. 32083461

[ref18] Chen Q.; Duan X.; Yu Y.; Ni R.; Song G.; Yang X.; Zhu L.; Zhong Y.; Zhang K.; Qu K.; et al. Target Functionalized Carbon Dot Nanozymes with Dual-Model Photoacoustic and Fluorescence Imaging for Visual Therapy in Atherosclerosis. Adv. Sci. 2023, 11, 2307441 10.1002/advs.202307441. PMC10853701 38145362

[ref19] Zhao Y.; Song M.; Yang X.; Yang J.; Du C.; Wang G.; Yi J.; Shan G.; Li D.; Liu L.; Yan D.; Li Y.; Liu X. Amorphous Ag2-xCuxS Quantum Dots: “All-in-One” Theranostic Nanomedicines for Near-Infrared Fluorescence/Photoacoustics Dual-Modal-Imaging-Guided Photothermal Therapy. Chem. Eng. J. 2020, 399, 125777 10.1016/j.cej.2020.125777.

[ref20] Xu Y.; Liang H.; Zeng Q.; He F.; Liu C.; Gai S.; Ding H.; Yang P. A Bubble-Enhanced Lanthanide-Doped Up/Down-Conversion Platform with Tumor Microenvironment Response for Dual-Modal Photoacoustic and Near-Infrared-II Fluorescence Imaging. J. Colloid Interface Sci. 2024, 659, 149–159. 10.1016/j.jcis.2023.12.088. 38159491

[ref21] Wang X.; Zhong X.; Li J.; Liu Z.; Cheng L. Inorganic Nanomaterials with Rapid Clearance for Biomedical Applications. Chem. Soc. Rev. 2021, 50, 8669–8742. 10.1039/D0CS00461H. 34156040

[ref22] Li Y.; Zheng X.; Chu Q. Bio-Based Nanomaterials for Cancer Therapy. Nano Today 2021, 38, 101134 10.1016/j.nantod.2021.101134.

[ref23] Wilhelm S.; Tavares A. J.; Dai Q.; Ohta S.; Audet J.; Dvorak H. F.; Chan W. C. Analysis of Nanoparticle Delivery to Tumours. Nat. Rev. Mater. 2016, 1 (5), 16014 10.1038/natrevmats.2016.14.

[ref24] Rennick J. J.; Johnston A. P. R.; Parton R. G. Key Principles and Methods for Studying the Endocytosis of Biological and Nanoparticle Therapeutics. Nat. Nanotechnol. 2021, 16, 266–276. 10.1038/s41565-021-00858-8. 33712737

[ref25] Mikušová V.; Mikuš P. Advances in Chitosan-Based Nanoparticles for Drug Delivery. Int. J. Mol. Sci. 2021, 22, 9652 10.3390/ijms22179652. 34502560 PMC8431817

[ref26] Dewhirst M.; Secomb T. Transport of Drugs from Blood Vessels to Tumour Tissue. Nat. Rev. Cancer 2017, 17, 738–750. 10.1038/nrc.2017.93. 29123246 PMC6371795

[ref27] de Lázaro I.; Mooney D. J. A Nanoparticle’s Pathway into Tumours. Nat. Mater. 2020, 19, 486–487. 10.1038/s41563-020-0669-9. 32332989

[ref28] Cai A.-Y.; Zhu Y.-J.; Qi C. Biodegradable Inorganic Nanostructured Biomaterials for Drug Delivery. Adv. Mater. Interfaces 2020, 7 (20), 2000819 10.1002/admi.202000819.

[ref29] Shao J.; Zhang J.; Jiang C.; Lin J.; Huang P. Biodegradable Titanium Nitride MXene Quantum Dots for Cancer Phototheranostics in NIR-I/II Biowindows. Chem. Eng. J. 2020, 400, 126009 10.1016/j.cej.2020.126009.

[ref30] He P.; Chen G.; Huang M.; Jing L.; Wu W.; Kuo H.-C.; Tu C.-C.; Chen S.-L. Biodegradable Germanium Nanoparticles as Contrast Agents for Near-Infrared-II Photoacoustic Imaging. Nanoscale 2023, 15, 11544–11559. 10.1039/D3NR01594G. 37366254

[ref31] Li M.; Liu Y.; Weigmann B. Biodegradable Polymeric Nanoparticles Loaded with Flavonoids: A Promising Therapy for Inflammatory Bowel Disease. Int. J. Mol. Sci. 2023, 24, 4454 10.3390/ijms24054454. 36901885 PMC10003013

[ref32] Zheng Z.; Jia Z.; Qu C.; Dai R.; Qin Y.; Rong S.; Liu Y.; Cheng Z.; Zhang R. Biodegradable Silica-Based Nanotheranostics for Precise MRI/NIR-II Fluorescence Imaging and Self-Reinforcing Antitumor Therapy. Small 2021, 17 (10), 2006508 10.1002/smll.202006508. 33569918

[ref33] Park Y.; Yoo J.; Kang M. H.; Kwon W.; Joo J. Photoluminescent and Biodegradable Porous Silicon Nanoparticles for Biomedical Imaging. J. Mater. Chem. B 2019, 7, 6271–6292. 10.1039/C9TB01042D. 31393507

[ref34] Fathi P.; Knox H. J.; Sar D.; Tripathi I.; Ostadhossein F.; Misra S. K.; Esch M. B.; Chan J.; Pan D. Biodegradable Biliverdin Nanoparticles for Efficient Photoacoustic Imaging. ACS Nano 2019, 13, 7690–7704. 10.1021/acsnano.9b01201. 31246412 PMC6903795

[ref35] Kang S. W.; Chung S. E.; Shin W. J.; Lee J.-H. Polypoidal Choroidal Vasculopathy and Late Geographic Hyperfluorescence on Indocyanine Green Angiography. Br. J. Ophthalmol. 2009, 93, 759–764. 10.1136/bjo.2008.145862. 19304584

[ref36] Tanaka E.; Chen F. Y.; Flaumenhaft R.; Graham G. J.; Laurence R. G.; Frangioni J. V. Real-Time Assessment of Cardiac Perfusion, Coronary Angiography, and Acute Intravascular Thrombi Using Dual-Channel Near-Infrared Fluorescence Imaging. J. Thorac. Cardiovasc. Surg. 2009, 138, 133–140. 10.1016/j.jtcvs.2008.09.082. 19577070 PMC2706783

[ref37] Xu Y.; Qi Z.; Luo Z.; Shen Z.; Li C.; Chen G.; Cai W.; Bao H.; Tu C.-C. Heat-Concentrating Solar Steam Generation and Salt Extraction Based on Water-Repellent Germanium Nanoparticles-Coated Oxidized Copper Foams. Sol. Energy Mater. Sol. Cells 2021, 230, 111191 10.1016/j.solmat.2021.111191.

[ref38] Zhao H.; Xue Z.; Wu X.; Wei Z.; Guo Q.; Xu M.; Qu C.; You C.; Mei Y.; Zhang M.; et al. Biodegradable Germanium Electronics for Integrated Biosensing of Physiological Signals. NPJ Flex. Electron. 2022, 6 (1), 63 10.1038/s41528-022-00196-2.

[ref39] Bukackova M.; Rusnok P.; Marsalek R. Mathematical Methods in the Calculation of the Zeta Potential of BSA. J. Solution Chem. 2018, 47, 1942–1952. 10.1007/s10953-018-0830-0.

[ref40] Chen G.; Wang L.; He P.; Su T.; Lai Q.; Kuo H.-C.; Wu W.; Chen S.-L.; Tu C.-C. Biodistributions and Imaging of Poly(ethylene glycol)-Conjugated Silicon Quantum Dot Nanoparticles in Osteosarcoma Models via Intravenous and Intratumoral Injections. ACS Appl. Bio Mater. 2023, 6, 4856–4866. 10.1021/acsabm.3c00595. 37843986

[ref41] Ge M.; Zong M.; Xu D.; Chen Z.; Yang J.; Yao H.; Wei C.; Chen Y.; Lin H.; Shi J. Freestanding Germanene Nanosheets for Rapid Degradation and Photothermal Conversion. Mater. Today Nano 2021, 15, 100119 10.1016/j.mtnano.2021.100119.

[ref42] Gagic M.; Kociova S.; Smerkova K.; Michalkova H.; Setka M.; Svec P.; Pribyl J.; Masilko J.; Balkova R.; Heger Z.; et al. One-Pot Synthesis of Natural Amine-Modified Biocompatible Carbon Quantum Dots with Antibacterial Activity. J. Colloid Interface Sci. 2020, 15, 30–48. 10.1016/j.jcis.2020.06.125. 32679365

[ref43] Yang L.; Huang B.; Chen F.; Jin J.; Qin Z.; Yang F.; Li Y.; Gu N. Indocyanine Green Assembled Nanobubbles with Enhanced Fluorescence and Photostability. Langmuir 2020, 36, 12983–12989. 10.1021/acs.langmuir.0c02288. 33085898

[ref44] Roper D. K.; Ahn W.; Hoepfner M. Microscale Heat Transfer Transduced by Surface Plasmon Resonant Gold Nanoparticles. J. Phys. Chem. C 2007, 111, 3636–3641. 10.1021/jp064341w. PMC2583113 19011696

[ref45] Belyaev I. B.; Zelepukin I. V.; Kotelnikova P. A.; Tikhonowski G. V.; Popov A. A.; Kapitannikova A. Y.; Barman J.; Kopylov A. N.; Bratashov D. N.; Prikhozhdenko E. S.; et al. Laser-Synthesized Germanium Nanoparticles as Biodegradable Material for Near-Infrared Photoacoustic Imaging and Cancer Phototherapy. Adv. Sci. 2024, 11 (20), 2307060 10.1002/advs.202307060. PMC11132077 38516744

[ref46] Lee C.; Kwon W.; Beack S.; Lee D.; Park Y.; Kim H.; Hahn S. K.; Rhee S. W.; Kim C. Biodegradable Nitrogen-Doped Carbon Nanodots for Non-Invasive Photoacoustic Imaging and Photothermal Therapy. Theranostics 2016, 6, 2196–2208. 10.7150/thno.16923. 27924157 PMC5135443

[ref47] Huang X.; Deng G.; Liao L.; Zhang W.; Guan G.; Zhou F.; Xiao Z.; Zou R.; Wang Q.; Hu J. CuCo_2_S_4_ Nanocrystals: A New Platform for Multimodal Imaging Guided Photothermal Therapy. Nanoscale 2017, 9, 2626–2632. 10.1039/C6NR09028A. 28155952

[ref48] Jiang X.; Du B.; Huang Y.; Yu M.; Zheng J. Cancer Photothermal Therapy with ICG-Conjugated Gold Nanoclusters. Bioconjugate Chem. 2020, 31, 1522–1528. 10.1021/acs.bioconjchem.0c00172. PMC8667163 32353229

