# Supplementary material for: Biodegradable ICG-Conjugated Germanium Nanoparticles for In Vivo Near-Infrared Dual-Modality Imaging and Photothermal Therapy
Source: ACS Appl Mater Interfaces. 2024 Oct 24;16(44):59752–64. doi: 10.1021/acsami.4c10800 (PMC11551961; doi:10.1021/acsami.4c10800)
Supplement: Supplementary file 1 — am4c10800_si_001.pdf [file am4c10800_si_001.pdf]

Supporting Information

Biodegradable ICG-Conjugated Germanium Nanoparticles for *In Vivo* Near-Infrared Dual-Modality Imaging and Photothermal Therapy

Guo Chen,[a] Pengbo He,[a] Cui Ma,[b] Jie Xu,[c] Taiyu Su,[a] Jingfei Wen,[a] Hao-Chung Kuo,[d] Lili Jing,[b] Sung-Liang Chen*[a,e,f,g] and Chang-Ching Tu*[a,d,h]*

[a]University of Michigan-Shanghai Jiao Tong University Joint Institute, Shanghai Jiao Tong University, Shanghai 200240, China

[b]Engineering Research Center of Cell & Therapeutic Antibody, Ministry of Education, and School of Pharmacy, Shanghai Jiao Tong University, Shanghai 200240, China

[c]School of Chemistry and Chemical Engineering, Shanghai Jiao Tong University, Shanghai 200240, China

[d]Semiconductor Research Center, Foxconn Research, Shenzhen 518109, China

[e]Institute of Medical Robotics, Shanghai Jiao Tong University, Shanghai 200240, China

[f]Engineering Research Center of Digital Medicine and Clinical Translation, Ministry of Education, Shanghai 200030, China

[g]State Key Laboratory of Advanced Optical Communication Systems and Networks, Shanghai Jiao Tong University, Shanghai 200240, China

[h]Department of Electrical Engineering, National Central University, Taiwan 320317, China

Corresponding Author

*Email: changching.tu@ee.ncu.edu.tw. Tel: +886-3-4227151 Ext. 34452

*Email: sungliang.chen@sjtu.edu.cn. Tel: +86-21-34206765 Ext. 4281

*Email: lilijing@sjtu.edu.cn Tel: +86-21-34204048

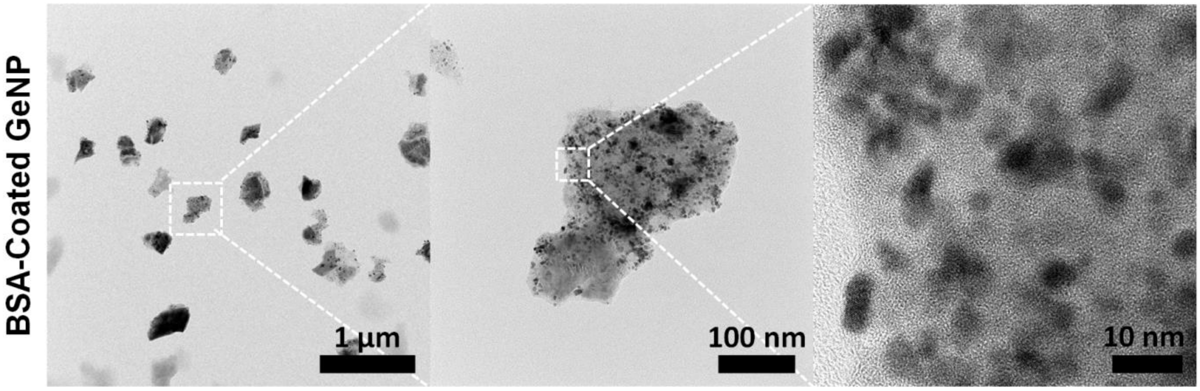

Figure S1. TEM images of the BSA-coated GeNPs.

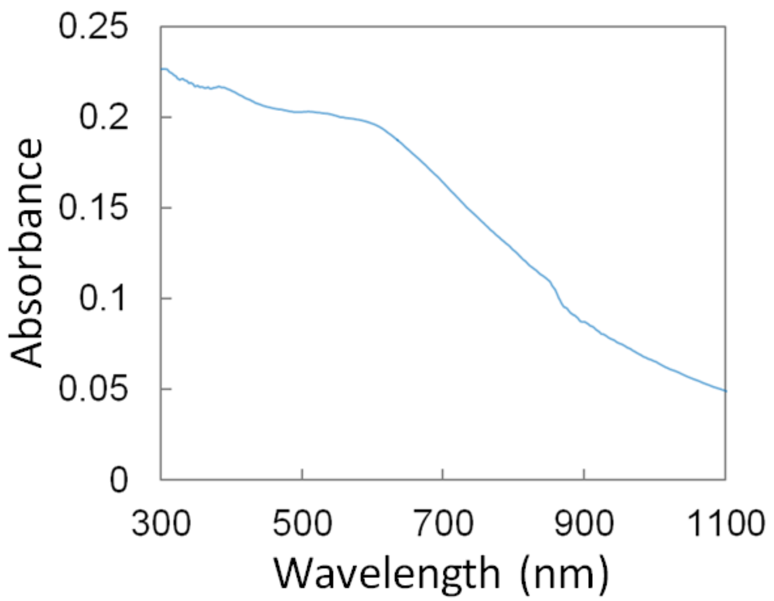

Figure S2. Absorbance spectrum of the BSA-coated GeNP suspension (25 µg mL^{-1} in 1× PBS). The absorbance spectrum was measured using a cuvette with an optical path equal to 1 cm.

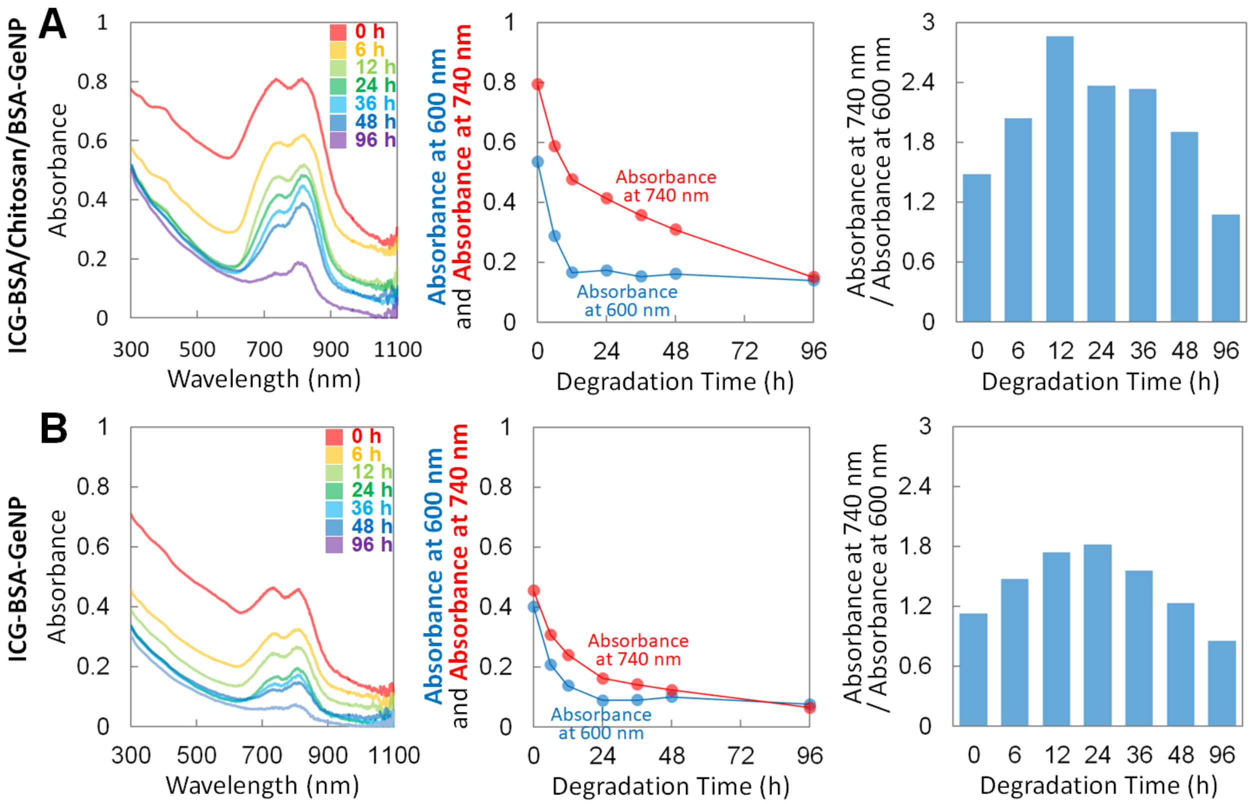

Figure S3. Absorbance spectra with different degradation times (left column), absorbance values at 600 nm and 740 nm (central column) and ratios of 740-nm absorbance to 600-nm absorbance as a function of degradation time (right column) for (A) the ICG-conjugated GeNPs with three-layered BSA/chitosan/BSA-coating and (B) the ICG-conjugated GeNPs with only one-layered BSA-coating.

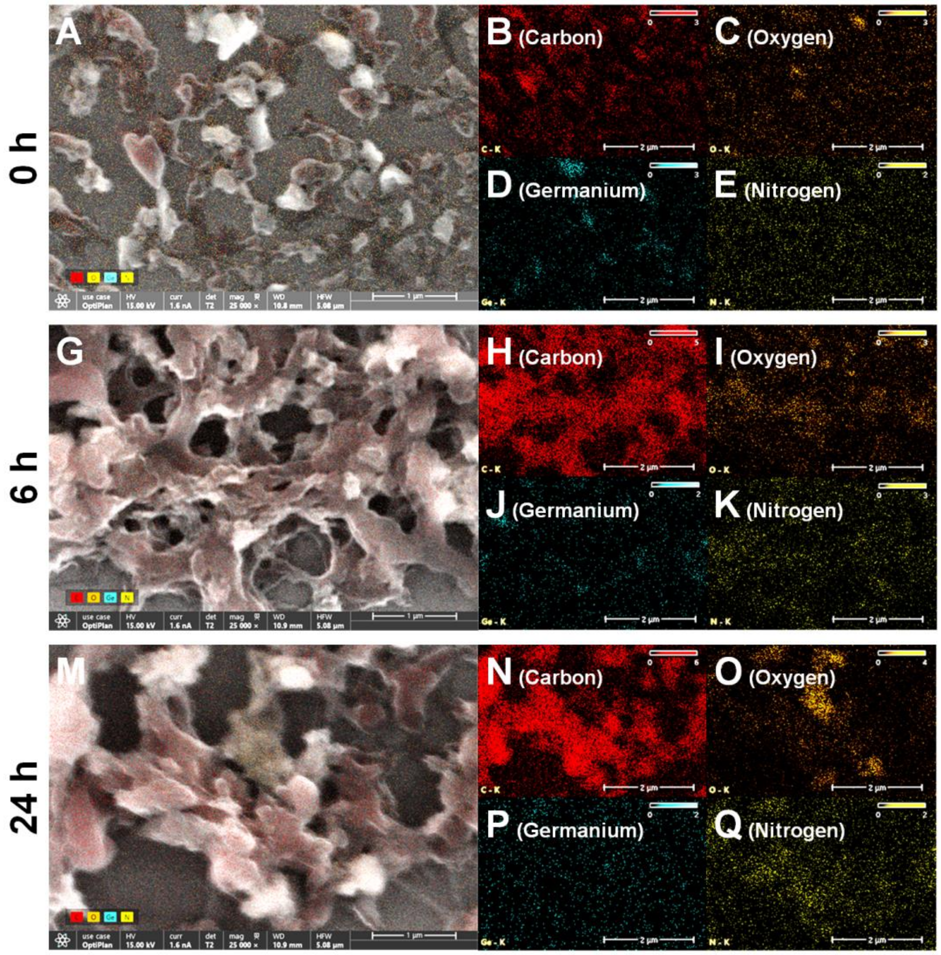

F

	Atomic %	Weight %
N	12.1	8.9
C	57.9	36.4
O	20.1	16.9
Ge	9.9	37.8

L

	Atomic %	Weight %
N	8.8	8.1
C	77.3	60.9
O	9.4	9.8
Ge	4.4	21.1

R

	Atomic %	Weight %
N	13.3	13.6
C	63.6	55.7
O	22.2	25.9
Ge	0.9	4.8

Figure S4. (A, G and M) SEM images, (B-E, H-K and N-Q) EDX elemental mapping images and (F, L and R) EDX elemental distributions of the ICG-conjugated GeNPs collected at 0 h, 6 h, and 24 h during the *in vitro* degradation process in 1× PBS at 37 ℃.

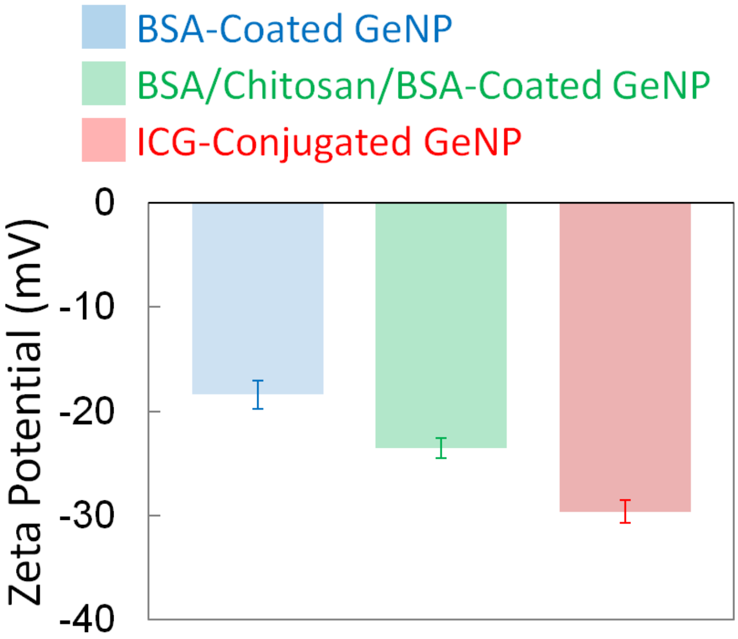

Figure S5. Zeta potential of the BSA-coated GeNPs, BSA/chitosan/BSA-coated GeNPs and ICG-conjugated GeNPs. Each sample was dispersed in pH 7 10 mM phosphate buffer with the concentration equal to 0.2 mg mL^{-1}. Each sample was measured thrice, with the average and standard deviation shown.

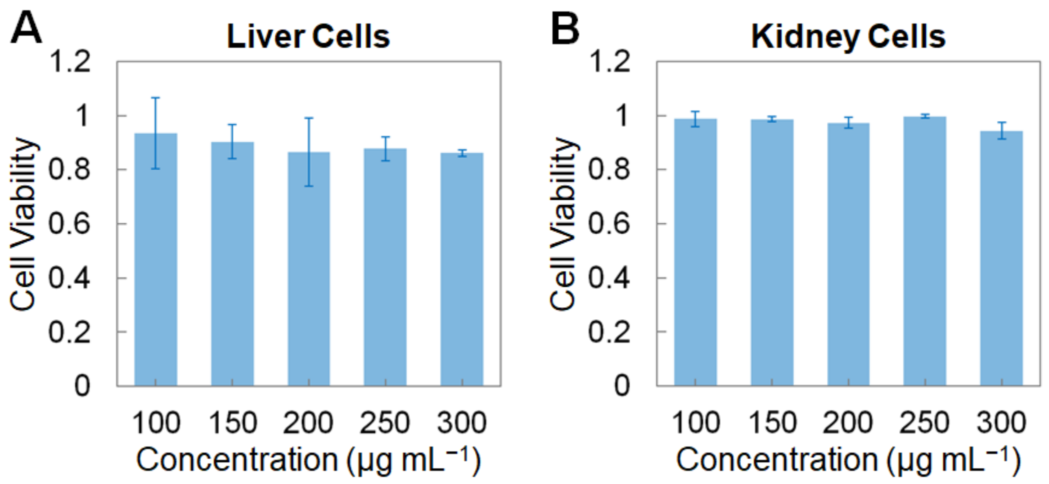

Figure S6. Cell viability assay for the normal cells of (A) liver and (B) kidney treated with the ICG-conjugated GeNPs. For a complete assay, three wells per concentration were adopted and the experiment was repeated three times. Each data point represents the mean value of n = 3 and the error bar is the standard deviation from the mean.

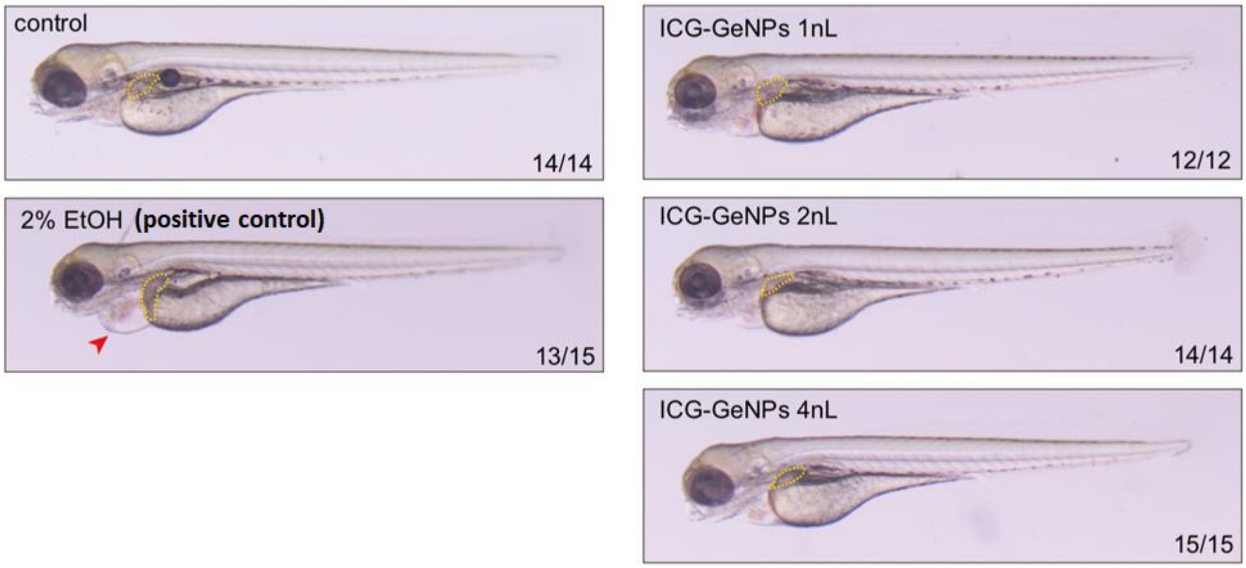

Figure S7. Toxicity test of the ICG-conjugated GeNPs on zebrafish embryos. The 24-hpf (hours post fertilization), wild-type zebrafish embryos were first dechorionated in about 0.05% pronase in E3 medium for 15 min. 1-phenyl 2-thiourea (PTU) was added to the medium to inhibit pigment formation. Subsequently, the zebrafish embryos were injected with 1 nL, 2 nL and 4 nL of the ICG-conjugated GeNPs (3 mg mL^{-1} in 1× PBS), respectively. For the positive control group, the zebrafish embryos were soaked in 2% EtOH for 48 h. For the control group, there was no treatment. After 48 h of incubation at 28.5℃ in dark, the zebrafish embryos were placed under a stereomicroscope (Olympus SZX16) for observation. For the positive control group, edema of pericardium (as indicated by the red arrow head) and swelling of liver (as indicated by the yellow dotted circle) were clearly identified. In contrast, the zebrafish embryos injected with the ICG-conjugated GeNPs showed no malformation in pericardium and liver. For each treatment condition, 10 to 20 zebrafish embryos per well on a 24-well plate were used. The ratio at the bottom right corner indicates the proportion of zebrafish embryos showing the result in the photograph.

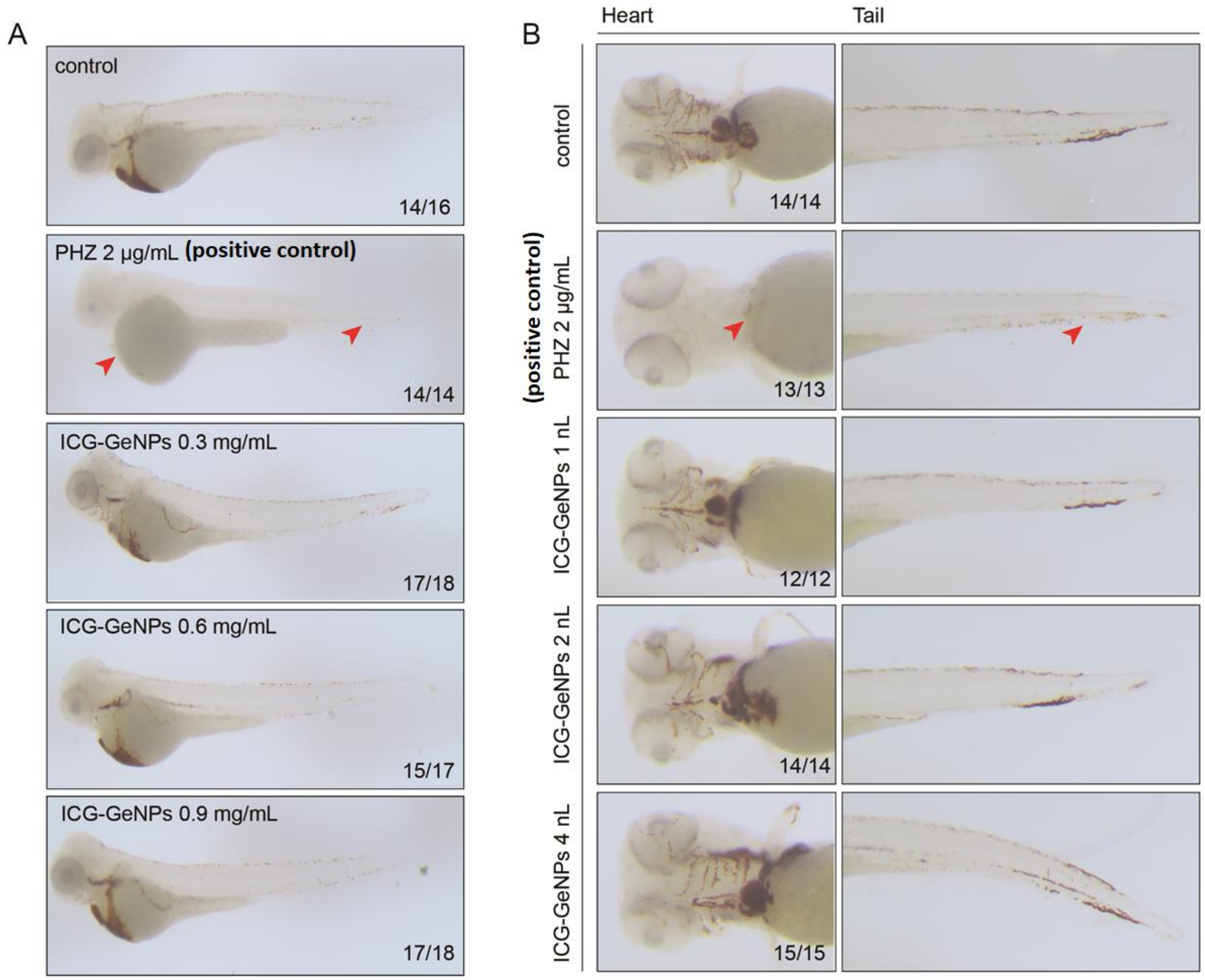

Figure S8. Hemolysis test of the ICG-conjugated GeNPs on zebrafish embryos. The 24-hpf (hours post fertilization), wild-type zebrafish embryos were first dechorionated in about 0.05% pronase in E3 medium for 15 min. 1-phenyl 2-thiourea (PTU) was added to the medium to inhibit pigment formation. Subsequently, the zebrafish embryos were (A) soaked in the ICG-conjugated GeNP suspensions in E3 medium with the concentrations equal to 0.3, 0.6 and 0.9 mg mL^{-1}, respectively, and (B) injected with 1 nL, 2 nL and 4 nL of the ICG-conjugated GeNP suspension in PBS with the concentration equal to 3 mg mL^{-1}, respectively. For the positive control group, the zebrafish embryos were soaked in 2 μg mL^{-1} phenylhydrazine (PHZ) for 24 h. For the control group, there was no treatment. After 48 h of incubation at 28.5℃ in dark, the

zebrafish embryos were dyed with benzidine and placed under a stereomicroscope (Olympus SZX16) for observation. For the positive control group, hemolysis (as indicated by the red arrow heads) was clearly identified. In contrast, the zebrafish embryos injected with the ICG-conjugated GeNPs showed no sign of hemolysis. For each treatment condition, 10 to 20 zebrafish embryos per well on a 24-well plate were used. The ratio at the bottom right corner indicates the proportion of zebrafish embryos showing the result in the photograph.

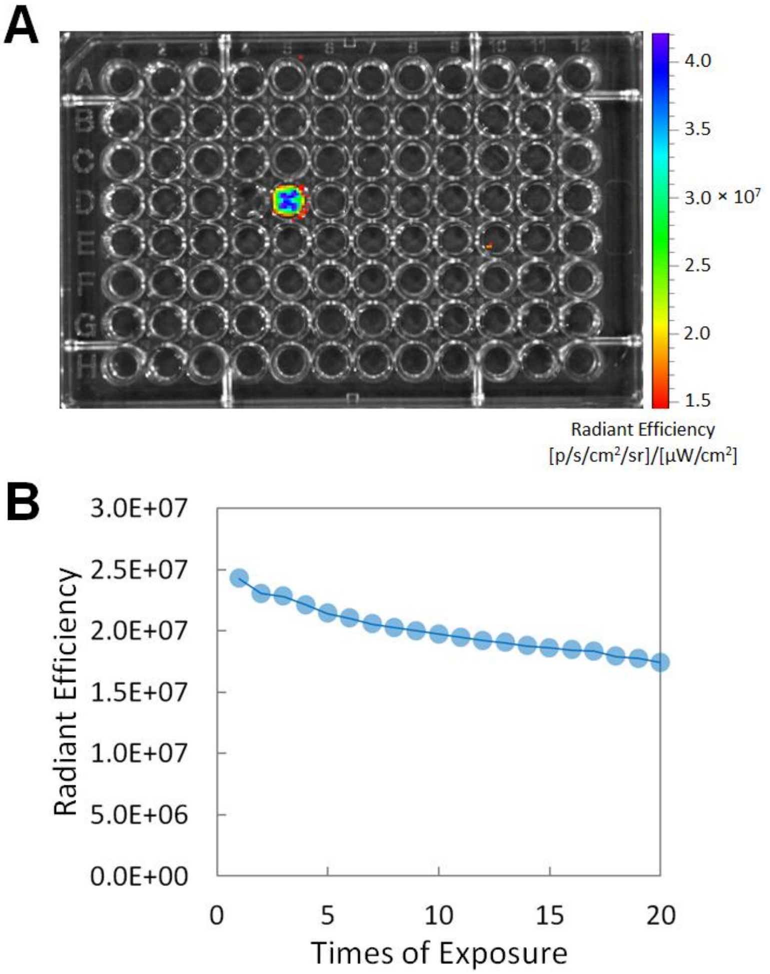

Figure S9. Photostability of the ICG-conjugated GeNPs under the IVIS imaging system. (A) Fluorescence image of the ICG-conjugated GeNPs (200 µL, 25 µg mL^{-1} in 1 × PBS) in a well of the 96-well culture plate. (B) Fluorescence intensity of the ICG-conjugated GeNPs as a function of times of exposure. Here the excitation wavelength was set at 745 nm and the emission wavelength at 840 nm. The exposure time was 15 s for each time.

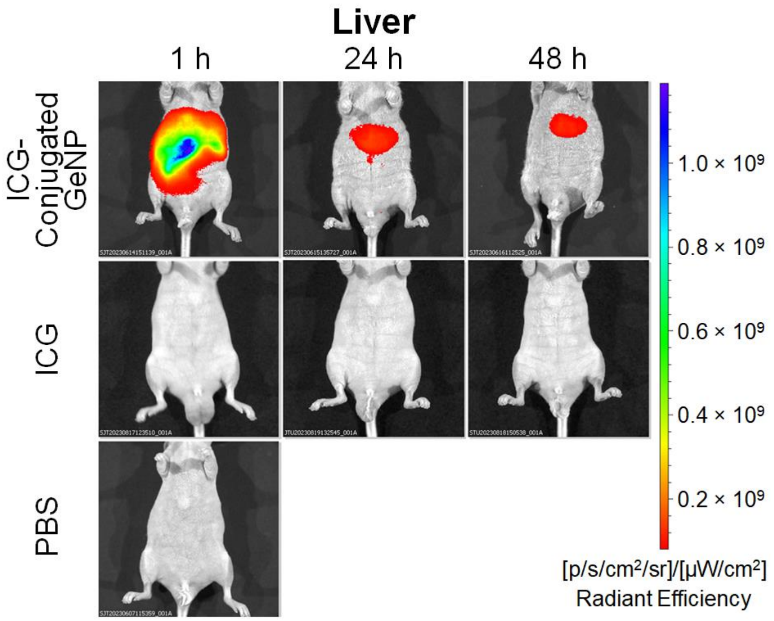

Figure S10. Time-dependent IVIS fluorescence images for highlighting the livers of the male nude mice intravenously injected with the ICG-conjugated GeNP suspension (150 µL, 3 mg mL^{-1} in 1× PBS), ICG solution (150 µL, 50 µg mL^{-1} in 1× PBS) and 1× PBS (150 µL, as the control group), respectively. Under the given range of the radiant efficiency colorbar, only the mouse injected with the ICG-conjugated GeNP suspension shows fluorescence signals at the liver. The excitation wavelength was set at 745 nm and the emission wavelength at 840 nm.

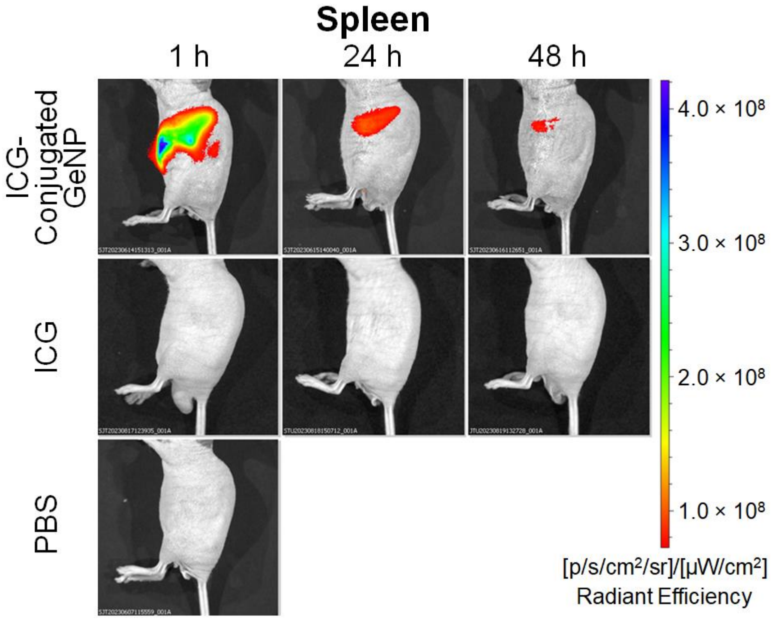

Figure S11. Time-dependent IVIS fluorescence images for highlighting the spleens of the male nude mice intravenously injected with the ICG-conjugated GeNP suspension (150 μL, 3 mg mL^{-1} in 1× PBS), ICG solution (150 μL, 50 μg mL^{-1} in 1× PBS) and 1× PBS (150 μL, as the control group), respectively. Under the given range of the radiant efficiency colorbar, only the mouse injected with the ICG-conjugated GeNP suspension shows fluorescence signals at the spleen. The excitation wavelength was set at 745 nm and the emission wavelength at 840 nm.

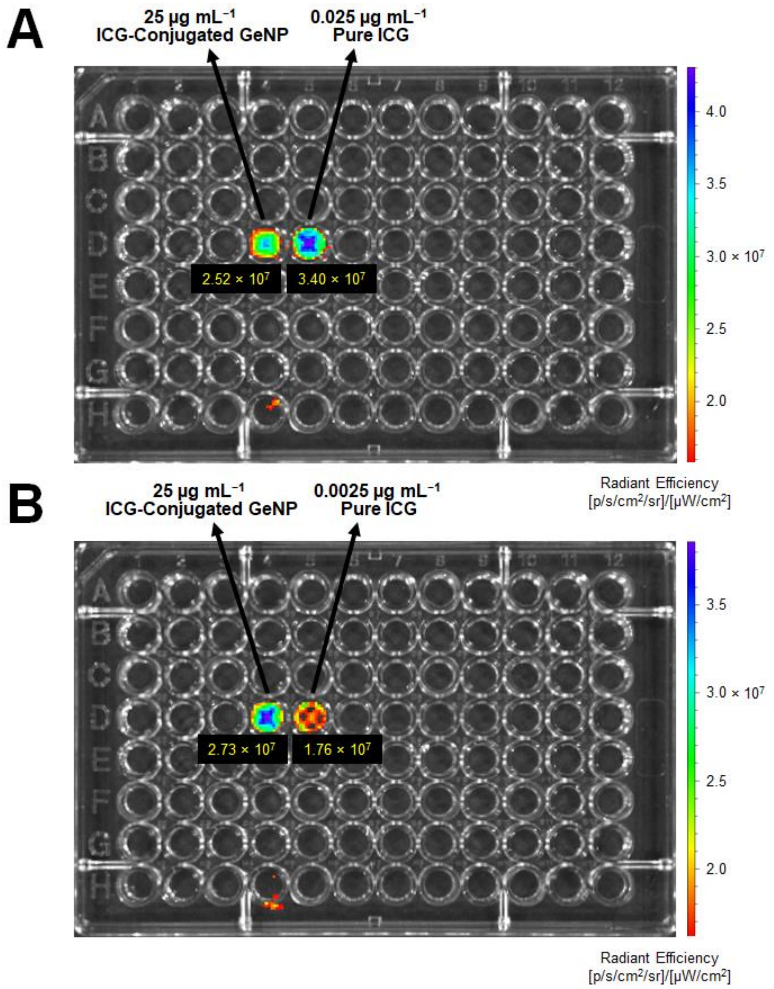

Figure S12. (A) Fluorescence image of the ICG-conjugated GeNPs (200 µL, 25 µg mL^{-1} in 1× PBS) and pure ICG (200 µL, 0.025 µg mL^{-1}) in two wells of the 96-well culture plate. (B) Fluorescence image of the ICG-conjugated GeNPs (200 µL, 25 µg mL^{-1} in 1× PBS) and pure ICG (200 µL, 0.0025 µg mL^{-1}) in two wells of the 96-well culture plate.

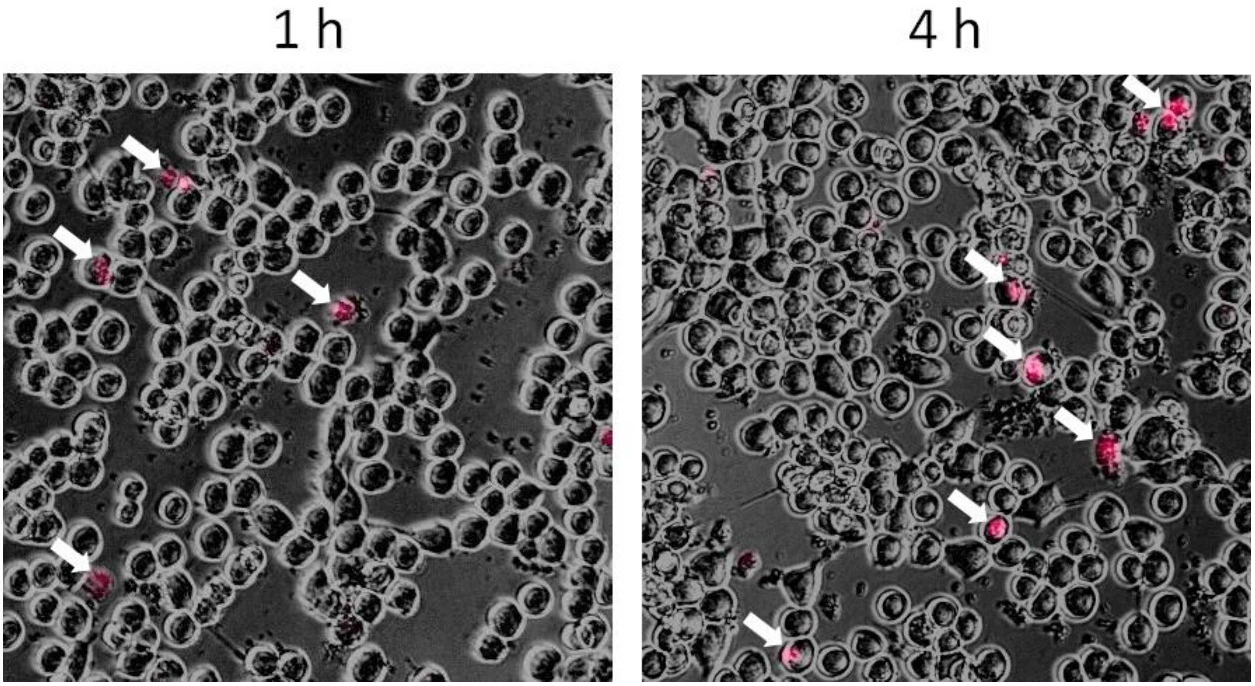

Figure S13. *In vitro* cellular uptake of the ICG-conjugated GeNPs. 4T1 cells were cultured in high glucose DMEM medium containing penicillin-streptomycin (100 unit L^{-1}) and 10% FBS in 5% CO_2 at 37℃. Before the cellular uptake experiment, the 4T1 cells were seeded in a 96-well plate (105 cells per well) and incubated for 24 h. During the experiment, the 4T1 cells were incubated with the ICG-conjugated GeNP suspension (0.15 mg mL^{-1}) for 1 and 4 hours at 37 ℃. After being thoroughly washed twice with PBS, the 4T1 cells were observed under the ImageXpress Micro Confocal High-Content Imaging System (Molecular Devices) with the excitation wavelength at 750 nm.

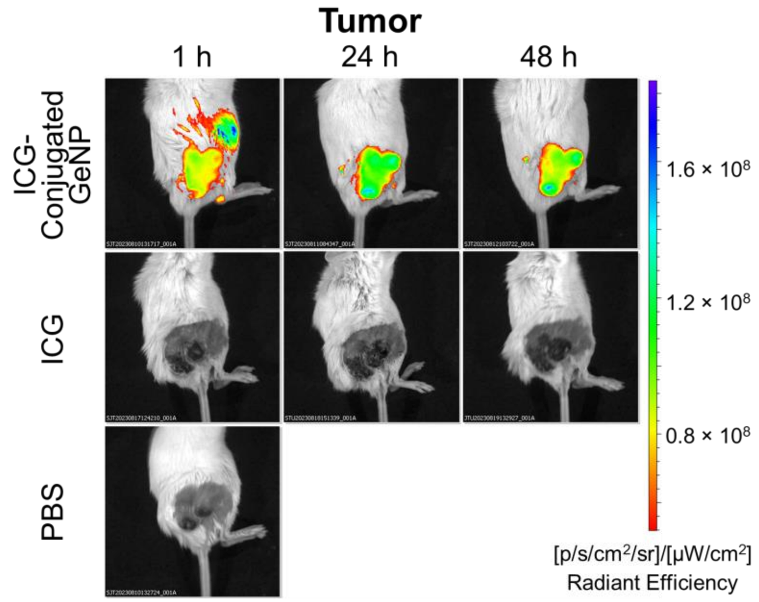

Figure S14. Time-dependent IVIS fluorescence images for highlighting the tumors of the 4T1 mouse models intravenously injected with the ICG-conjugated GeNP suspension (150 μL, 3 mg mL^{-1} in 1× PBS), ICG solution (150 μL, 50 μg mL^{-1} in 1× PBS) and 1× PBS (150 μL, as the control group), respectively. Under the given range of the radiant efficiency colorbar, the mouse model injected with the ICG-conjugated GeNP suspension shows fluorescence signals at both the liver and tumor at 1 h after the injection, but the liver fluorescence signal disappears at 24 h after the injection. In comparison, the tumor model injected with the ICG solution shows no fluorescence at the tumor. The excitation wavelength was set at 745 nm and emission wavelength at 840 nm.

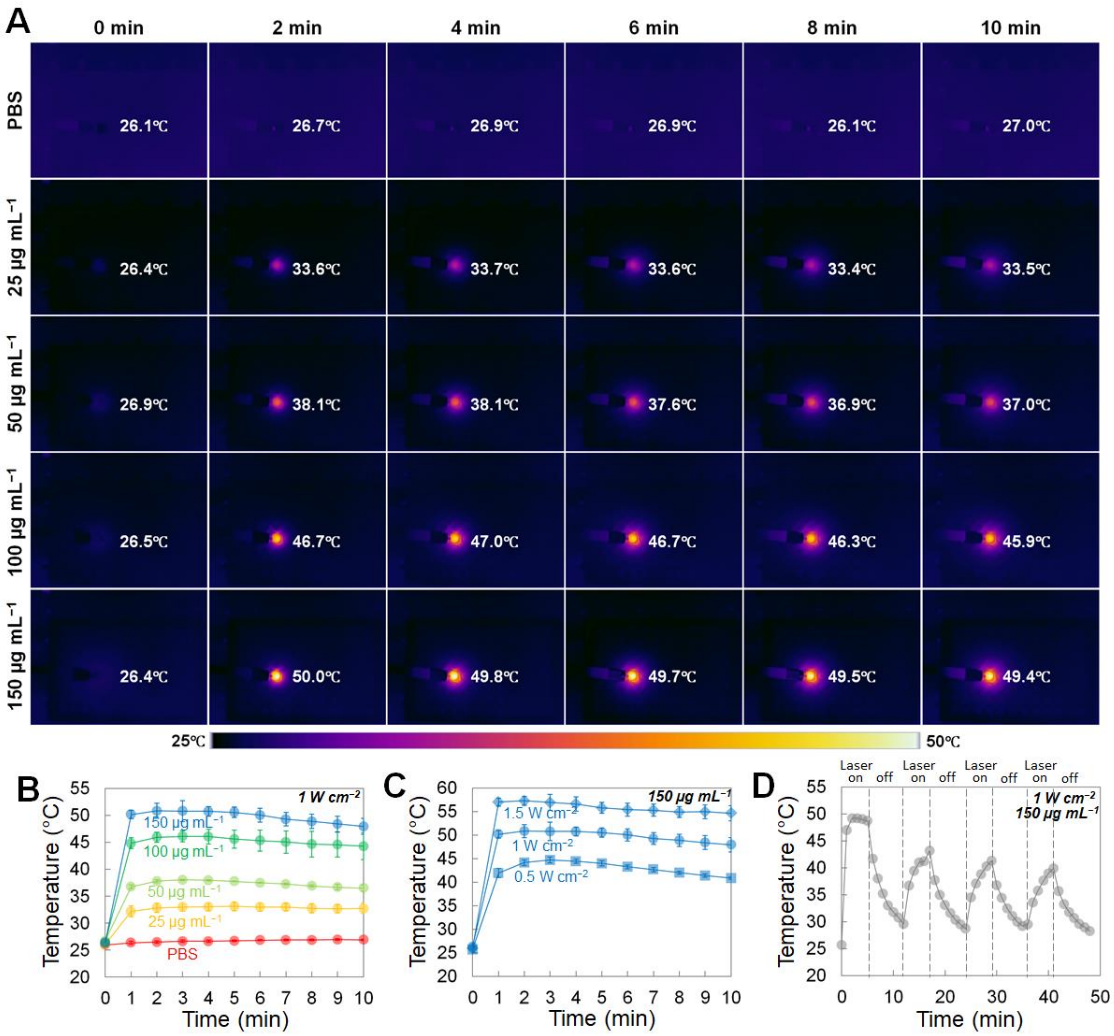

Figure S15. *In vitro* photothermal properties of the ICG-conjugated GeNPs. (A) Thermographic maps of PBS and the ICG-conjugated GeNP suspensions of different concentrations (25, 50, 100 and 150μg mL^{-1}) loaded into different wells of a 96-well culture plate under 808 nm laser irradiation (1 W cm^{-2}) for 10 min. (B) Time-resolved temperatures of PBS and the ICG-conjugated GeNP suspensions of different concentrations under 808 nm laser irradiation (1 W cm^{-2}) for 0 to 10 min. (C) Time-resolved temperatures of the ICG-conjugated GeNP suspension (150μg mL^{-1}) under 808 nm laser irradiation with different intensities (0.5, 1 and 1.5 W cm^{-2}) for 0 to 10 min. (D) Time-resolved temperature of the ICG-conjugated GeNP suspension (150μg mL^{-1}) in four heating/cooling cycles.

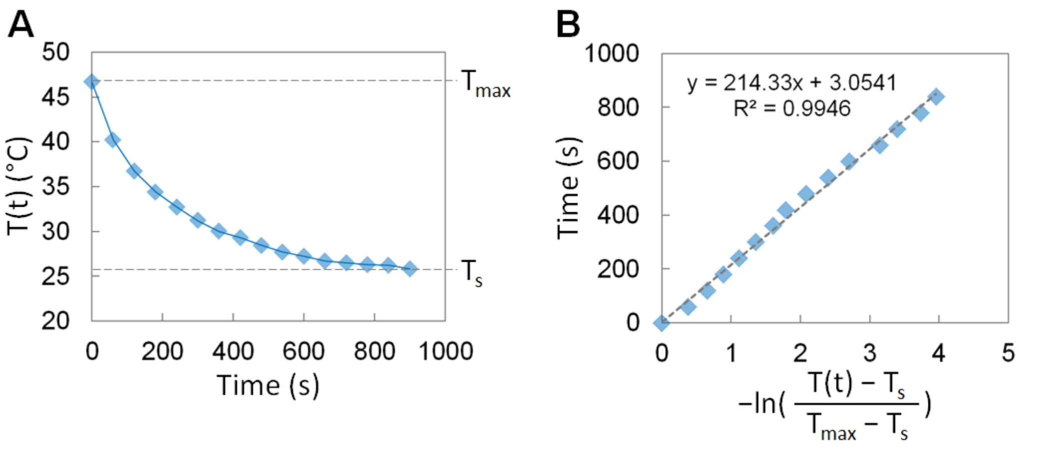

$$\textbf{Photothermal Conversion Efficiency } (\eta) = \frac{m \times C \times (\Delta T_{max}^{GeNP} - \Delta T_{max}^{PBS})}{\tau_c \times P \times (1 - 10^{-A_{808}})} = 51\%$$

$m = 0.3$ g

$C = 4.2$ J g^{-1} °C^{-1}

$\Delta T_{max}^{GeNP} = 50.9 - 26.3 = 24.6$°C (from blue line in Figure S14B)

$\Delta T_{max}^{PBS} = 26.9 - 26 = 0.9$°C (from red line in Figure S14B)

$\tau_c = 214.33$ s (from the fitted line slope in Figure S15B)

$P = 1$ W cm^{-2} × π × 0.32^2 cm^2 = 0.32 W (well radius = 0.32 cm)

$A_{808} = 0.8$

Figure S16. Estimation of the photothermal conversion efficiency of the ICG-conjugated GeNPs. (A) Temperature cooling curve of the ICG-conjugated GeNP suspension (300 μL, 150μg mL^{-1} and loaded in a well of the 96-well plate) after 808 nm laser irradiation (1 W cm^{-2}) for 10 min. (B) Graph for calculating the cooling temperature coefficient, τ_c, which is equal to the slope of the fitted line. The photothermal conversion efficiency can be calculated by using the equation shown above, where m and C represent the mass and heat capacity of the ICG-conjugated GeNP suspension, ΔT_{max}^{GeNP} and ΔT_{max}^{PBS} are the maximum elevated temperatures of the ICG-conjugated GeNP suspension and PBS under the laser irradiation, which can be obtained from the blue and red lines in Figure S15B, respectively, P is the irradiation power and A_{808} is the absorbance of the ICG-conjugated GeNP suspension in the well.

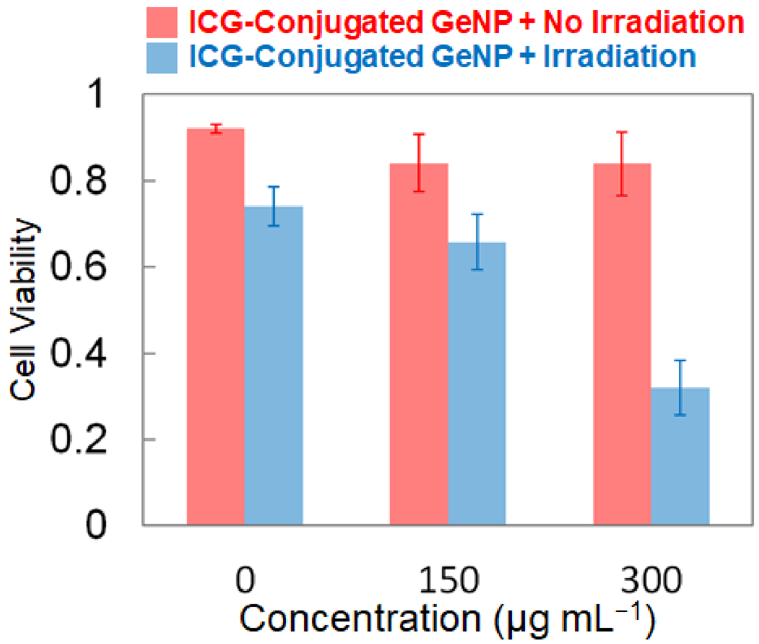

Figure S17. Cell viability assay for the 4T1 cell line treated by the ICG-conjugated GeNPs with or without 808 nm laser irradiation (1 W cm^{-2}) for 10 min. For a complete assay, three wells per concentration were adopted and the assay was repeated three times. Each data point represents the mean value of n = 3 and the error bar is the standard deviation from the mean.

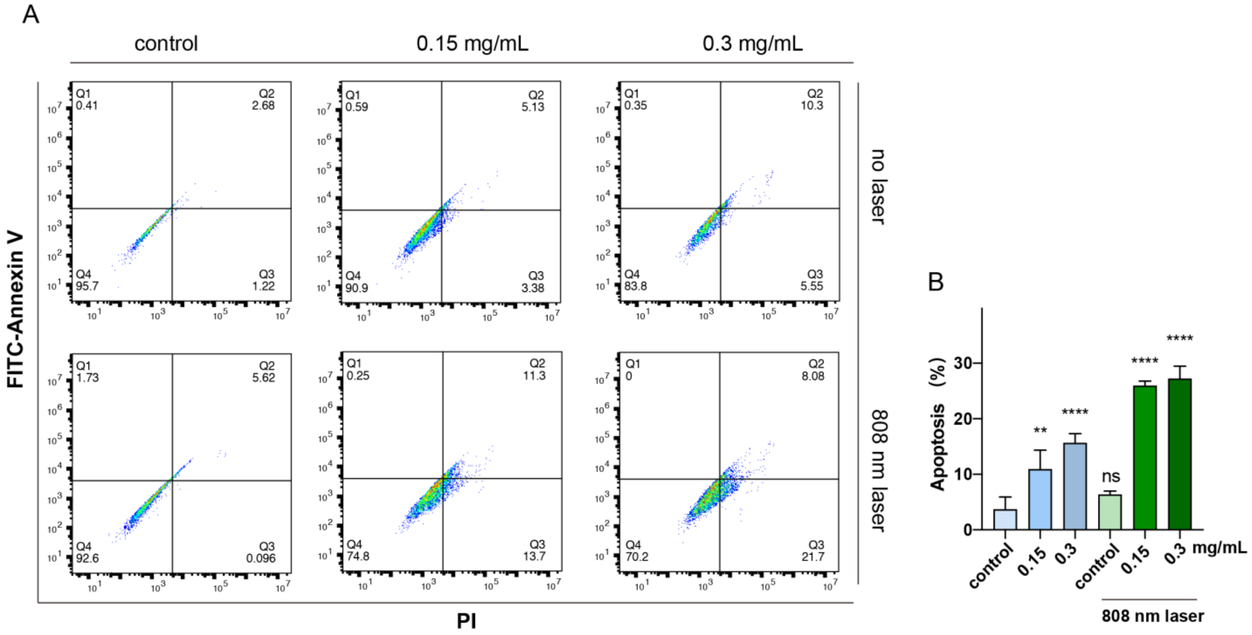

Figure S18. Apoptosis assay of the ICG-conjugated GeNPs. (A) Representative flow cytometry dot-plots of 4T1 cells treated with the ICG-conjugated GeNPs with or without 808 nm laser irradiation (1 W cm^{-2}) for 10 min. Here viable cells are located in the lower left quadrant (FITC−/PI−), early apoptotic cells in the upper right quadrant (FITC+/PI+) and late apoptotic cells in the lower right quadrant (FITC−/PI+). (B) Proportions of 4T1 cells in the early and late apoptosis quadrants as determined by the flow cytometry dot-plots in (A). In general, the proportion of apoptotic cells increased as the ICG-conjugated GeNP concentration increased. Furthermore, the laser irradiation also increased the apoptosis rate. The data are presented as the mean ± standard deviation of three independent experiments. *$P < 0.05$. The experimental procedure of the apoptosis assay is briefly described as follows. First, 4T1 cells (5 × 10^{4}) were seeded in a 24-well plate. For the photothermal treatment, the cells were treated with the ICG-conjugated GeNPs (0, 150 and 300 μg mL^{-1}) at 37℃ for 2 h with or without the 808 nm laser irradiation (1 W cm^{-2}) for 10 min. Subsequently, the cells were collected, washed with 1× PBS and re-suspended in 500 μL of binding buffer, followed by staining with propidium iodide (PI,

50 μg mL^{-1}) and FITC-conjugated Annexin V for 15 min at room temperature in dark. Lastly, the cells were analyzed using a flow cytometer. The flow cytometry data were analyzed using the FlowJo software. For a complete assay, three wells per concentration were adopted and the assay was repeated three times.

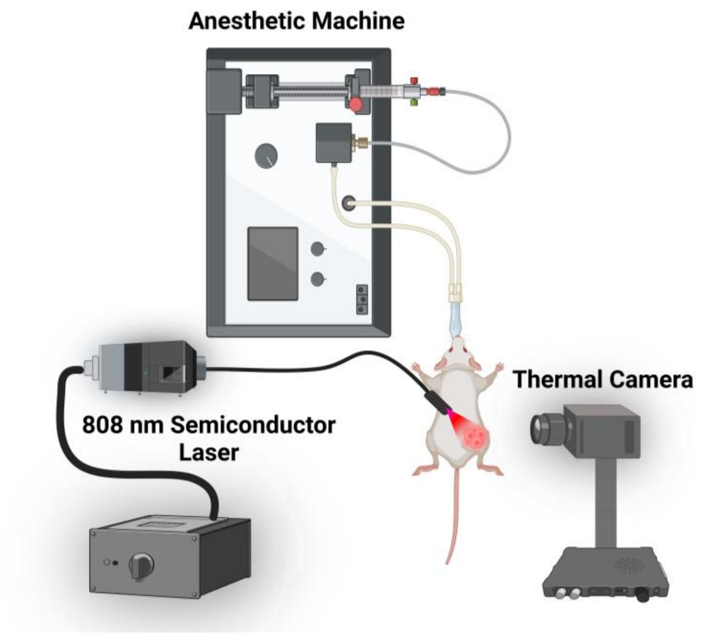

Figure S19. Schematic diagram of the photothermal treatment. The image created by BioRender.com with permission.

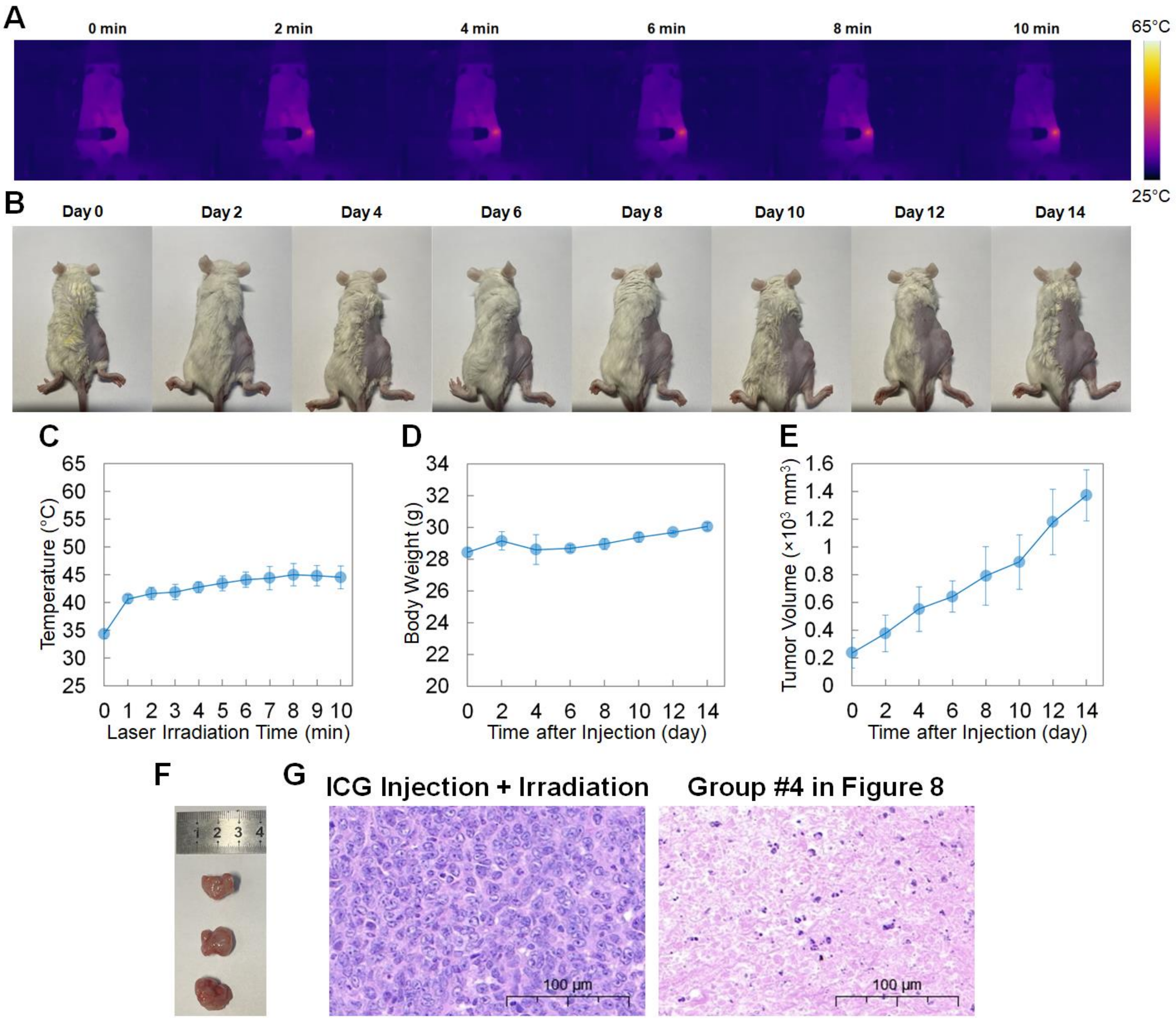

Figure S20. (A and C) Time-dependent infrared thermographic maps and tumor temperatures of the 4T1 tumor models injected with pure ICG solution (150 μL, 50 μg mL^{-1} in 1× PBS) during the photothermal treatment. (B, D and E) Time-dependent photographs, body weights and tumor volumes of the 4T1 tumor models injected with pure ICG solution (150 μL, 50 μg mL^{-1} in 1× PBS) after the photothermal treatment. The data were recorded every 2 days for a total of 14 days. Each data point in Figure S20C, S20D and S20E represents the mean value of n = 3 (i.e., three mice for each data point) and the error bar is the standard deviation from the mean. (F and G) Photographs and representative H&E stained image of the tumors dissected from mice injected with pure ICG solution at the end of the 14-day observation period.

Experimental Section

Synthesis of the ICG-Conjugated GeNPs: Fragments of n-type (111) crystalline Ge wafer (about 0.6 g) were placed in a zirconia milling jar containing about 80 g of zirconia beads and 25 mL of isopropanol (IPA). By high-energy ball milling (MITR QM-QX0.4L), the Ge wafers were first pulverized into coarse powder using 10-mm zirconia beads for 3 h and then further ground into nanoparticles using 3-mm zirconia beads for 20 h. The resulting GeNPs were etched in 25 to 28 wt% NH_4OH for 2 h, followed by fast centrifugation (12k rcf for 10 min) and dispersion in IPA by sonication. The suspension was further applied with slow centrifugation (3k rcf for 1 min) to precipitate large particles, and then the remaining small-sized GeNPs in the supernatant were collected by fast centrifugation, followed by etching with 8 wt% HF for removing surface oxide. Subsequently, the HF-etched GeNPs were dispersed in deoxygenated neat UDA, along with catalytic amount of chloroplatinic acid, for a heat-induced hydrosilylation reaction at 130℃ for more than 20 h. The UDA-functionalized GeNPs were precipitated by fast centrifugation with the addition of hexane as the antisolvent, washed several times in tetrahydrofuran (THF) and dispersed in dimethyl sulfoxide (DMSO) by sonication. For the conjugation with BSA, the carboxylic acids of the UDA ligands were first activated by 100 mM 1-ethyl-3-(3-dimethylaminopropyl) carbodiimide hydrochloride (EDC·HCl) and 50 mM N-hydroxysulfosuccinimide (Sulfo-NHS) in DMSO at 60℃ for 48 h, followed by centrifugal precipitation, dispersion in 20 mg mL^{-1} BSA in pH 7.4 0.1 M PB for 3 h and washing several times in pH 7 0.1 M PB. To increase the primary amine binding sites for the conjugation with ICG, the BSA-coated GeNPs were sequentially dispersed by sonication in chitosan solution (3 mg mL^{-1} in 1 % v/v acetic acid with the pH adjusted to 5) for 1 h and then BSA solution (10 mg mL^{-1} in water) for 2 h. The chitosan before dilution has a viscosity equal to 100 to 200

millipascal-second (mPa.s). The chitosan and BSA molecules were physically adsorbed onto the GeNP surfaces. The binding forces between the chitosan and BSA molecules should be primarily electrostatic interaction, as evidenced by the zeta potential change in Figure S5. Finally, the BSA/chitosan/BSA-coated GeNPs were treated with NHS-functionalized ICG (0.05 mg mL^{-1} in water) for 12 h. For preventing the degradation of GeNPs in water, the reaction temperature was kept at 4℃. The resulting ICG-conjugated GeNPs were washed with water several times to remove the freestanding ICG, re-dispersed in 1× PBS and stored at 4℃ for the following experiments.

Animal Models: All animal experiments were performed in accordance with the guidelines approved by the Institutional Animal Care and Use Committee of Shanghai Jiao Tong University. For the body weight analysis (Figure 2B), histological imaging on major organs (Figure 2C and 2D) and *in vivo* biodistribution analysis (Figure 4), male nude mice (about 25 g) obtained from the Shanghai Laboratory Animal Research Center were used. For the *ex vivo* biodistribution analysis (Figure 5 and 6), male BALB/c mice obtained from the Shanghai Laboratory Animal Research Center were used. For the *in vivo* dual-modality imaging of tumors (Figure 7) and photothermal therapy on tumors (Figure 8), 4T1 tumor models were used, which were prepared as follows. First, 4T1 cells (2×10^7) were subcutaneously injected at the right hindlimbs of the male BALB/c mice aged 5 to 6 weeks. At 10 day after the inoculation, the subcutaneous tumors grew to a volume around 150 mm^3, and the models were ready for the experiments.

Cell Viability Assay: For the assays, 4T1 cells were seeded in a 96-well culture plate, with 8×10^3 cells in 200 μL of Gibco RPMI 1640 medium per well. After incubation at 37°C and 5% CO_2 for 24 h, the medium was replaced by 200 μL of fresh medium containing the ICG-conjugated GeNPs of varying concentrations (50, 100 150, 200, 250 and 300 μg mL^{-1}). Here the ICG-

conjugated GeNP suspensions after full degradation for 96 h at 37°C were used for treating the cells, for preventing the partial-degraded GeNP residues from adhering to the cells and causing inaccurate reading of absorption. After incubation for another 48 h, each well was washed two times with fresh medium and then added with 10 μL CCK8 solution. After incubation at 37°C for another 2 h, using an ELISA microplate reader, the cell viability (%) was determined by comparing the absorbance at 450 nm of the well treated with the ICG-conjugated GeNPs to that of the control well not receiving the treatment. A complete assay (three wells per concentration of the ICG-conjugated GeNPs) was repeated three times and the results were averaged.

Body Weight Analysis and Histological Imaging on Major Organs: After intravenous tail vein injections of the ICG-conjugated GeNPs (150 μL, 3 mg mL^{-1} in 1× PBS) and 1× PBS only (150 μL, as the control group), respectively, the weights of the male nude mice were recorded daily for 14 days. Subsequently, the mice were sacrificed, and their major organs (liver, spleen, kidney, lung and heart) were dissected, fixed in 4% paraformaldehyde, embedded in melted paraffin wax and cut into 4-μm slices. The slices were stained with H&E and examined under a microscope.

Biodistribution by In Vivo Dual-Modality Imaging: After intravenous tail vein injections of the ICG-conjugated GeNPs (150 μL, 3 mg mL^{-1} in 1× PBS) and 1× PBS only (150 μL, as the control group), respectively, the male nude mice were imaged at different time points (1 h, 24 h and 48 h). The mice were anesthetized by isoflurane during the image acquisition. For each imaging time point, fluorescence imaging was conducted using the IVIS imaging system (PerkinElmer Spectrum), for which the excitation wavelength was set at 745 nm and the

emission wavelength at 840 nm. Then the mouse was moved to the PACT system (VEVO LAZR-X) for PA imaging, for which the excitation wavelength was also set at 745 nm.

Biodistribution by Ex Vivo Dual-Modality Imaging: After intravenous tail vein injections of the ICG-conjugated GeNPs (150 μL, 3 mg mL^{-1} in 1× PBS) and 1× PBS only (150 μL, as the control group), respectively, the male BALB/c mice were sacrificed at different time points (1 h, 24 h and 48 h). Their major organs (liver, spleen, kidney, lung and heart) were dissected and immediately subjected to fluorescence imaging using the IVIS imaging system, for which the excitation wavelength was set at 745 nm and the emission wavelength at 840 nm. Then the major organs were moved to the PACT system (VEVO LAZR-X) for PA imaging, for which the excitation wavelength was also set at 745 nm.

In Vivo Dual-Modality Imaging on 4T1 Tumor Models: After intravenous tail vein injections of the ICG-conjugated GeNPs (150 μL, 3 mg mL^{-1} in 1× PBS) and 1× PBS only (150 μL, as the control group), respectively, the 4T1 tumor models were imaged at different time points (1 h, 24 h and 48 h). The mice were anesthetized by isoflurane during image acquisition. For each imaging time point, fluorescence imaging was conducted using the IVIS imaging system, for which the excitation wavelength was set at 745 nm and the emission wavelength at 840 nm. Then the mice were moved to the PACT system (VEVO LAZR-X) for PA imaging, for which the excitation wavelength was set at 745 nm.

Characterizations: TEM images were obtained by JEOL JEM-2100. DLS particle size distribution was obtained by Malvern Panalytical Zetasizer Nano S. Absorbance spectra were obtained by Thermo Scientific Genesys 10S UV-Vis. PL spectra were obtained by Edinburgh FLS1000 photoluminescence spectrometer. FTIR spectra were obtained by Thermo Scientific

Nicolet 6700 with the attenuated total reflection module attached. Fluorescence imaging was obtained by IVIS Spectrum imaging system (PerkinElmer). Photoacoustic imaging was obtained by PACT system (VEVO LAZR-X).
